# HMMR triggers immune evasion of hepatocellular carcinoma by inactivation of phagocyte killing

**DOI:** 10.1126/sciadv.adl6083

**Published:** 2024-06-05

**Authors:** Hong Wu, Yiqiang Liu, Qianshi Liu, Zhaoshen Li, Yejian Wan, Chenhui Cao, Binghuo Wu, MingXin Liu, Renchuan Liang, Lanlin Hu, Wenyi Zhang, Mei Lan, Quan Yao, Hang Zhou, Haitao Lan, Liang Chen, Yu Zhang, Xia Zhang, Xiu-Wu Bian, Chuan Xu

**Affiliations:** ^1^ Department of Oncology & Cancer Institute, Sichuan Academy of Medical Sciences, Sichuan Provincial People’s Hospital, University of Electronic Science and Technology of China, Chengdu 610072, P. R. China.; ^2^ Department of Laboratory Medicine and Sichuan Provincial Key Laboratory for Human Disease Gene Study, Sichuan Provincial People's Hospital, University of Electronic Science and Technology of China, Chengdu 610072, P. R. China.; ^3^ Institute of Pathology and Southwest Cancer Center, Southwest Hospital, Third Military Medical University (Army Medical University), Chongqing 400038, P. R. China.; ^4^ Yu-Yue Pathology Scientific Research Center, Chongqing 400039, P. R. China.; ^5^ Sichuan Cancer Hospital & Institute, Sichuan Cancer Center, School of Medicine, University of Electronic Science and Technology of China, Chengdu 610072, P. R. China.; ^6^ Department of Cardiology, The First Affiliated Hospital of USTC, Division of Life Science and Medicine, University of Science and Technology of China, Hefei 230027, P. R. China.; ^7^ The Department of Hepatobiliary and Pancreatic Surgery, Sichuan Academy of Medical Sciences, Sichuan Provincial People’s Hospital, University of Electronic Science and Technology of China, Chengdu 610072, P. R. China.; ^8^ Jinfeng Laboratory, Chongqing 400039, P. R. China.

## Abstract

Hepatocellular carcinoma (HCC) acquires an immunosuppressive microenvironment, leading to unbeneficial therapeutic outcomes. Hyaluronan-mediated motility receptor (HMMR) plays a crucial role in tumor progression. Here, we found that aberrant expression of HMMR could be a predictive biomarker for the immune suppressive microenvironment of HCC, but the mechanism remains unclear. We established an HMMR^−/−^ liver cancer mouse model to elucidate the HMMR-mediated mechanism of the dysregulated “don't eat me” signal. HMMR knockout inhibited liver cancer growth and induced phagocytosis. HMMR^high^ liver cancer cells escaped from phagocytosis via sustaining CD47 signaling. Patients with HMMR^high^CD47^high^ expression showed a worse prognosis than those with HMMR^low^CD47^low^ expression. HMMR formed a complex with FAK/SRC in the cytoplasm to activate NF-κB signaling, which could be independent of membrane interaction with CD44. Notably, targeting HMMR could enhance anti–PD-1 treatment efficiency by recruiting CD8^+^ T cells. Overall, our data revealed a regulatory mechanism of the “don't eat me” signal and knockdown of HMMR for enhancing anti–PD-1 treatment.

## Teaser

This work revealed a regulatory mechanism of the “don't eat me” signal, and targeting HMMR could enhance treatment efficacy.

## INTRODUCTION

Hepatocellular carcinoma (HCC), a primary liver cancer transformed from hepatocytes, is the third leading cause of cancer-related death globally, accounting for 80% of primary liver malignancies (*1*). First-line therapy suffers from poor response that leads to a high mortality rate with a 5-year survival rate below 12% (*2*). The immunosuppressive microenvironment with massive tumor-associated macrophages (TAMs) infiltration is recognized as the primary factor for the treatment failure (*3*); thus, reducing the TAMs to enhance the antitumor activities serves as an important therapeutic strategy for HCC treatments. To date, lines of evidence revealed that cell surface molecules transmit an antiphagocytic signal, known as the “don’t eat me” signal, to the innate immune system, such as CD47 (*4*), SIRPG (*5*), programmed cell death protein 1 (PD-1) (*6*), the beta-2 microglobulin subunit of the primary histocompatibility class I complex (B2M) (*7*), and CD24 (*8*). However, the complexity of tumor cells and the poor therapeutic effect of antiphagocytosis have suggested the presence of additional, as yet unknown, “don’t eat me” signals.

Hyaluronan-mediated motility receptor (HMMR; also known as RHAMM) is an oncogene that correlates with aggressive tumor growth and poor patient survival in multiple cancer types such as lung cancer (*9*), breast cancer (*10*), and prostate cancer (*11*). Despite previous studies depicting the evolutionarily conserved role of HMMR in hyaluronan binding (*12*), the pervasive existence of HMMR in multiple species that lack hyaluronan strongly indicates that it might also serve multifunctional roles in physiopathological regulations (*13*–*15*). For instance, HMMR serves as a binding partner for spindle assembly factors—such as TPX2, DYNLL1, and CHICA/FAM83D—to regulate the assembly, stability, and positioning of spindle microtubules during mitosis and meiosis (*16*). These protein complexes are critical in correctly orienting the mitotic spindle and establishing the cell division axis that regulates the metastasis of cancer cells (*17*). Recent study also indicated that the level of HMMR expression in lung adenocarcinoma (LUAD) was notably linked to the infiltration of neutrophils, CD8^+^ T cells and CD4^+^ T cells (*18*). However, the immunoregulatory role and the underlying mechanism of HMMR in antiphagocytic of macrophages remains elusive that requires deeper understanding.

In this study, we uncovered that HMMR facilitated the antiphagocytic efficiency via the HMMR-CD47 axis of liver cancer cells. HMMR could form a complex with FAK/SRC in the cytoplasm independent of membrane expressed CD44 to help immune evasion from phagocyte killing. Loss of HMMR could probably brake the “don't eat me” signal and sensitize immune checkpoint blockade inhibition for cancer immunotherapy in patients with HCC.

## RESULTS

### HMMR^−/−^ inhibits DEN-induced liver cancer growth

**Fig. 1.**
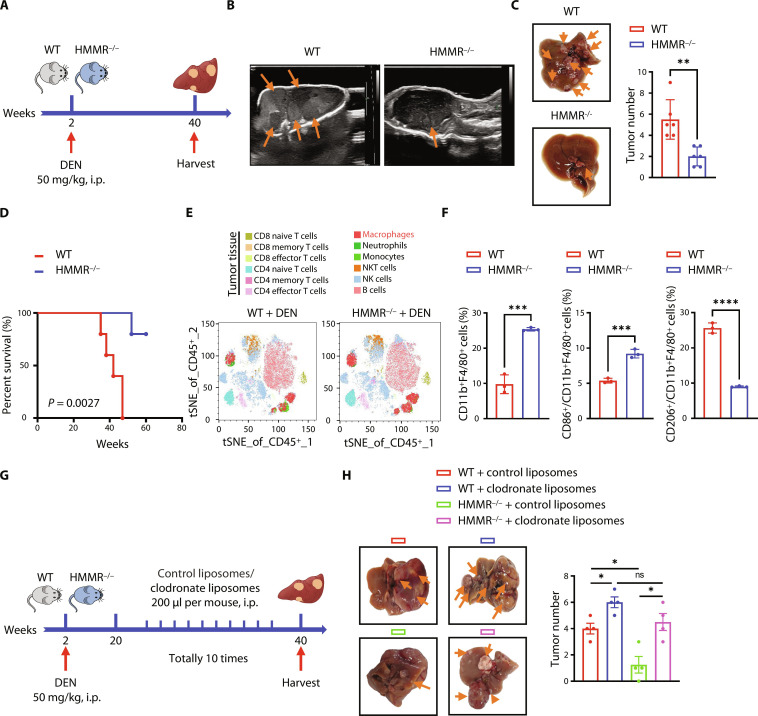
HMMR^−/−^ inhibits liver cancer growth by enhancing the infiltration of macrophages. (**A**) Schematic showing the construction process of DEN-induced HCC mice model. Two-week-old mice were treated with a single injection of DEN (50 mg/kg) and euthanized at 40 weeks for further analysis. (**B**) Representative ultrasound images showing the liver tumorigenesis in DEN-induced WT and HMMR^−/−^ mice. (**C**) Representative images and statistical analysis of the liver tumor numbers in DEN-induced WT and HMMR^−/−^ mice (*n* = 6). (**D**) Overall survival of DEN-induced WT and HMMR^−/−^ mice. (**E**) Multicolor flow cytometry analyzed the infiltration of intratumoral immune cells in DEN-induced liver cancer. (**F**) Flow cytometry analyzed the infiltration of intratumoral immune cells, the percent of CD11b^+^F4/80^+^ cells, the ratio of CD86^+^ cells in CD11b^+^F4/80^+^, and the ratio of CD206^+^ cells in CD11b^+^F4/80^+^ cells. (**G**) Schematic showing the construction process of DEN-induced HCC mice model and macrophage depletion with clodronate liposomes. (**H**) Representative images and statistical analysis of the liver tumorigenesis in DEN-induced WT and HMMR^−/−^ mice with control liposomes or clodronate liposomes treatment (*n* = 4). All experiments were carried out in triplicate, and the data are presented as the means ± SEM. The *P* values are calculated by log-rank test, unpaired, two-tailed Student’s *t* test, or one-way ANOVA. *P < 0.05, **P < 0.01, ***P < 0.001, and ****P < 0.0001. ns, nonsignificant.

To investigate the functional role of the potential driver gene in HCC, we detected HMMR expression in tumor tissues and adjacent normal tissues. Results from our center, The Cancer Genome Atlas (TCGA), and Gene Expression Omnibus (GEO) database demonstrated that HMMR was highly expressed in tumor tissues and associated with the poor prognosis of patients with HCC (fig. S1, A to J). Multivariate Cox regression analysis revealed that HMMR could be deemed as an independent predictive indicator for the poor prognosis of patients with HCC (fig. S1K). To reveal the underlying functional role of HMMR, we constructed a transgenic mouse model and then applied diethylnitrosamine (DEN) to induce liver cancer (Fig. 1A and fig. S2A). Results demonstrated that HMMR knockout (HMMR^−/−^) prolonged the survival of tumor-bearing mice and reduced tumor growth (Fig. 1, B to D). We then collected the liver cancer tissues from WT and HMMR^−/−^ mice to detect the accumulation of immune cells, including T lymphocytes (CD4^+^, CD8^+^), macrophages, CD20^+^ B lymphocytes, natural killer cells, and CD66b^+^ neutrophils in HCC tissues. The tumor tissues of HMMR^−/−^ mice have a higher proportion of macrophages (Fig. 1E). We further confirmed that tumor tissues from HMMR^−/−^ mice were infiltrated more by CD11b^+^F4/80^+^ and CD86^+^CD11b^+^F4/80^+^ (M1) macrophages (Fig. 1F). To explore whether tumor suppression partially accounts for the cross-talk between macrophages and cancer cells in HMMR^−/−^ mice, we performed in vivo macrophage depletion assay using clodronate liposomes and found that depletion of macrophages could rescue the reduction of tumorigenicity in HMMR^−/−^ mice, supporting the notion that HMMR promotes tumorigenesis through regulating the role of macrophages (Fig. 1, G and H, and fig. S2B). Our results further demonstrated that HMMR had no influence on the proliferation of liver cancer cells in vitro (fig. S2, C and D). These results together suggest that the functional role of HMMR in tumor growth partially requires the participation of immune cells such as macrophages.

### HMMR orchestrates the infiltration of intratumoral immune cells to facilitate the antiphagocytosis of tumor cells

**Fig. 2.**
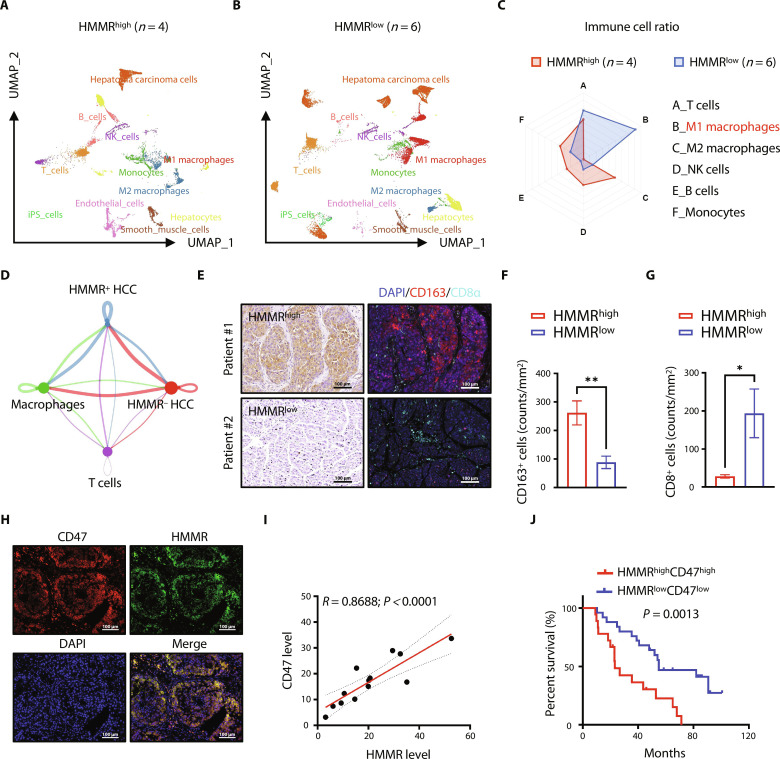
HMMR^low^-expressed cancer cells facilitate the intratumoral infiltration of macrophages. (**A** and **B**) UMAP plot, showing the annotation and color codes for cell types in HMMR^high^ (*n* = 4) and HMMR^low^ expressed (*n* = 6) patients. The single-cell RNA sequencing data were accessed from the GEO database (GSE149614). (**C**) Cell ratio of different cell clusters between HMMR^high^- and HMMR^low^-expressed patients. (**D**) Networks visualizing potential specific interactions between pairs of two cell populations, in which circle sizes were proportional to the count of cells in each cell group, and edge width means the communication probability. The network layout was set to the force-directed layout. (**E**) Representative images of immunohistochemical staining (HMMR) and polychromatic immunofluorescence staining of corresponding regions in the serial section. Scale bars, 100 μm. (**F** and **G**) Statistical analysis of CD163^+^ macrophages and CD8^+^ T cells between HMMR^high^- and HMMR^low^-expressed patients. (**H** and **I**) Immunofluorescence staining of HMMR and CD47 in patients with HCC (H) and coexpression analysis of the two proteins (I). Scale bars, 100 μm. (**J**) Kaplan-Meier analysis assessed the prognostic value of combining HMMR and CD47 expression in 80 HCC samples. The *P* values are calculated by simple linear regression, log-rank test for survival, and unpaired, two-tailed Student’s *t* test. *P < 0.05 and **P < 0.01..

To explore the functional role of HMMR in regulating tumor immune microenvironment, we collected public single-cell HCC data (GSE149614) and used uniform manifold approximation and projection (UMAP) for dimension reduction. The patients with HCC were divided into two groups based on the expression level of HMMR, including four HMMR^high^-expressed patients and six HMMR^low^-expressed patients. We conducted UMAP analyses stratified by HMMR level and compared the proportions of cell types, including macrophages, T cells, B cells, etc., between the two groups. The results indicated that patients with HMMR^low^ exhibited higher infiltration of M1 macrophages and T cells (Fig. 2, A to C, and fig. S3A). We studied signaling communication of HMMR^high^- or HMMR^low^-expressed cancer cells and macrophages or T cells. CellChat analysis identified that HMMR^low^-expressed cancer cells communicated more intensively with macrophages than T cells (Fig. 2D). To further validate the role of HMMR in regulating the immune microenvironment, we used multiplex immunofluorescence staining to characterize the intratumoral infiltration of immune cells. Less infiltration of CD163^+^ macrophages and more infiltration of CD8^+^ T cells in the tumor immune microenvironment were found in HMMR^low^-expressed patients with HCC (Fig. 2, E to G). We performed a systematic bioinformatics analysis of gene-gene interaction networks in the STRING database using the cBio Cancer Genomics Portal (http://cbioportal.org). Results showed that HMMR and CD47 were located in a network containing 24 nodes, suggesting a potential link between HMMR and CD47 signaling (fig. S3B). We further detected the expression of HMMR and CD47 expression by immunofluorescence staining, coexpression analysis showed that the two proteins had a high positive coexpression (*R* = 0.8688, *P* < 0.0001) (Fig. 2, H and I). The single-cell RNA sequencing data further demonstrated that HMMR expression in cancer cells was positively correlated with CD47 expression in HCC tissues (fig. S3, C to E). We then analyzed the prognostic value of HMMR and CD47 expression in 80 patients with HCC to determine the potential clinical significance. Results showed that the patients with HMMR^high^CD47^high^ expression have the worse prognosis than those with HMMR^low^CD47^low^ expression (Fig. 2J). Conclusively, our results show that HMMR expression is associated with the infiltration of immune cells and may participate in regulating the phagocytosis of cancer cells.

### The HMMR-CD47 axis facilitates immune escape from phagocytosis

**Fig. 3.**
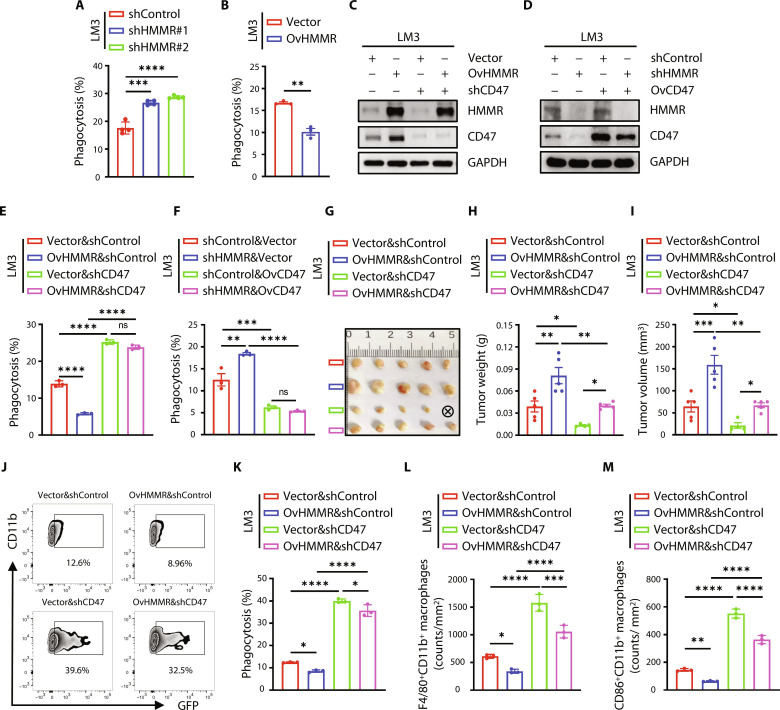
HMMR facilitates the antiphagocytosis through CD47 signaling. (**A** and **B**) Phagocytosis analysis of shControl and shHMMR (A) and Vector and OvHMMR (B) cancer cells. All experiments were carried out at least in triplicate, and the data were presented as means ± SEM. (**C**) Immunoblotting analysis of indicated proteins in Vector and OvHMMR LM3 cells with or without CD47 knockdown. (**D**) Immunoblotting analysis of indicated proteins in shControl and shHMMR LM3 cells with or without CD47 overexpression. (**E**) Flow cytometric analyses for the phagocytosis of Vector and OvHMMR LM3 cells with or without CD47 knockdown (2 × 10^5^ cells per tube) by F4/80 marked BMDMs (2 × 10^5^ cells per tube). (**F**) Phagocytosis of shControl and shHMMR LM3 cells with or without CD47 overexpression. (**G** to **I**) Tumor image (G), tumor weight (H), and tumor volume (I) of Balb/c nude mice inoculated with Vector and OvHMMR LM3 cells with or without CD47 knockdown (1 × 10^6^ inoculated cells per mice, *n* = 5 mice per group). (**J** and **K**) Phagocytosis of indicated LM3 xenografts was represented by the percentage of GFP^+^F4/80^+^CD11b^+^ cells in total F4/80^+^CD11b^+^ cells. (**L** and **M**) Immunofluorescence staining and statistical analysis of F4/80^+^CD11b^+^ and CD86^+^CD11b^+^ macrophages of indicated groups. All experiments were carried out at least in triplicate, and the data are presented as means ± SEM. The *P* values are calculated using two-tailed Student’s *t* test or two-way ANOVA. *P < 0.05, **P < 0.01, ***P < 0.001, and ****P < 0.0001. ns, nonsignificant.

To investigate the functional role of HMMR in antiphagocytic efficiency, we conducted phagocytosis assays by coculturing cancer cells and bone marrow–derived macrophage (BMDM)–derived M1 macrophages. Results showed that knockdown of HMMR enhanced phagocytosis of cancer cells, while overexpression of HMMR inhibited phagocytosis by flow cytometry (Fig. 3, A and B, and fig. S4, A to D). We cocultured PKH26-labeled macrophages and carboxyfluorescein diacetate succinimidyl ester–labeled cancer cells to test phagocytosis by confocal microscopy. Results showed that overexpression of HMMR in Huh7 cells could decrease the phagocytosis of macrophages, while knockdown of HMMR could increase the phagocytosis (fig. S4, E and F). In light of our observation that HMMR has a potential role in regulating phagocytosis, we asked whether it transmitted the signal by regulating CD47 expression. Following this idea, we constructed HMMR overexpression and CD47 knockdown or HMMR knockdown and CD47-overexpressed liver cancer cells (Fig. 3, C and D, and fig. S4, G and H). We observed that HMMR overexpression inhibited the phagocytosis, while the knockdown of CD47 reversed the attenuated phagocytosis upon HMMR overexpression (Fig. 3E and fig. S4I). Similarly, overexpression of CD47 reversed the heightened phagocytosis in HMMR-knockdown cancer cells (Fig. 3F and fig. S4J). We next sought to determine whether CD47 signaling is central to the ability of HMMR to regulate tumorigenesis in vivo. We observed that knockdown of CD47 inhibited tumor growth and abrogated the tumor-promoting effect upon HMMR overexpression (Fig. 3, G to I). To investigate whether the functional role of HMMR/CD47 axis in promoting tumor development was resulted partly from inhibition of phagocytosis, we used green fluorescent protein (GFP)–labeled LM3 cells to assess their engulfment by macrophages in vivo. We identified phagocytic events by flow cytometry, assessing the percentage of CD11b^+^F4/80^+^GFP^+^ cells among total CD11b^+^F4/80^+^ murine macrophages. We observed that CD47 knockdown rescued the inhibited phagocytosis of HMMR overexpression in LM3 cells in vivo (Fig. 3, J and K). In addition, we performed immunofluorescence staining to costain CD11b^+^F4/80^+^ and CD86^+^CD11b^+^ macrophages in the four specified groups. Results demonstrated that OvHMMR led to a decrease in the infiltration of CD11b^+^F4/80^+^ and CD86^+^CD11b^+^ macrophages, whereas shCD47 increased macrophage infiltration (Fig. 3, L and M). Similarly, CD47 restoration sufficed to rescue the defect in tumorigenesis caused by HMMR knockdown (fig. S4, K to M). Notably, CD47 restoration abrogated the heightened phagocytosis upon HMMR deficiency (fig. S4N), indicating the role of HMMR/CD47 axis in suppressing phagocytosis in vivo. Thus, CD47 expression is required for HMMR to promote phagocytosis of liver cancer cells.

### HMMR activates FAK/SRC signaling independent of CD44 to sustain CD47 expression

**Fig. 4.**
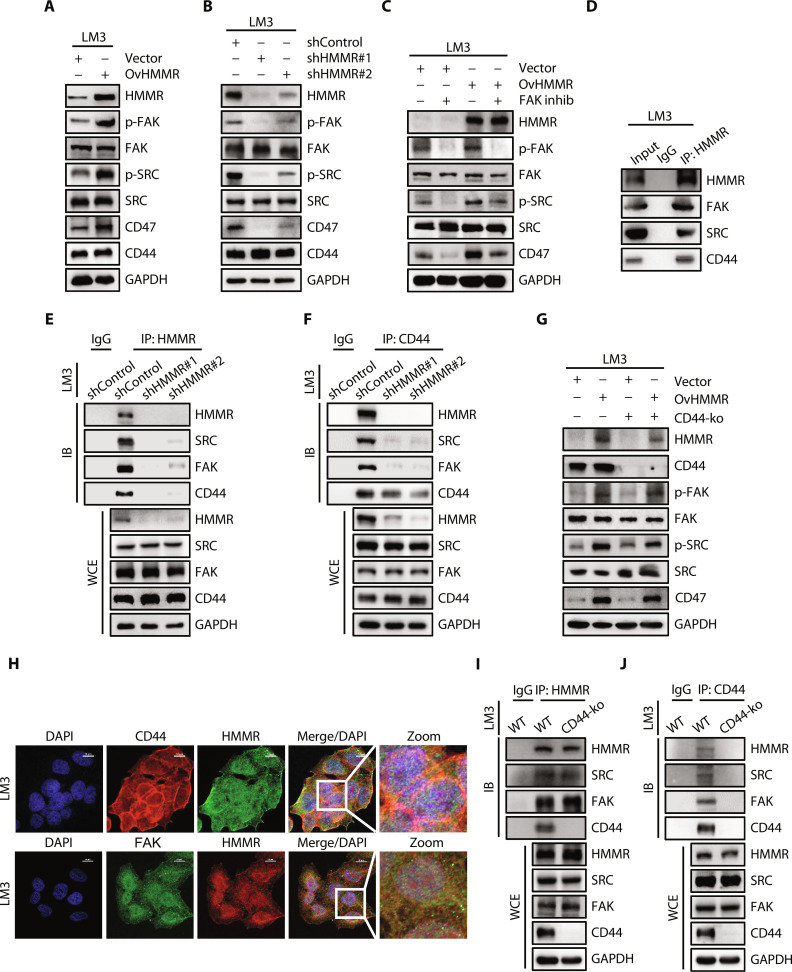
HMMR activates FAK/SRC signaling independent of CD44 to sustain CD47 expression in patients with HCC. (**A**) Immunoblotting analysis of indicated proteins in Vector and OvHMMR LM3 cells. (**B**) Immunoblotting analysis of indicated proteins in shControl and shHMMR LM3 cells. (**C**) Immunoblotting analysis of indicated proteins in Vector and OvHMMR LM3 cells with or without FAK inhibitor (10 μM) treatment for 24 hours. (**D**) Immunoprecipitation analysis of the interaction between HMMR, FAK, SRC, and CD44 in LM3 cells. (**E** and **F**) Immunoprecipitation analysis of the interaction between HMMR, FAK, SRC, and CD44 in shControl and shHMMR LM3 cells. (**G**) Immunoblotting analysis of indicated proteins in Vector and OvHMMR LM3 cells with or without CD44-ko. (**H**) Representative images showing the co-localization of HMMR, CD44, and FAK detecting by immunofluorescence. Nuclei were counterstained with 4′,6-diamidino-2-phenylindole (DAPI) (blue). Scale bars, 10 μm. (**I** and **J**) Immunoprecipitation analysis of the interaction between HMMR, FAK, SRC, and CD44 in control and CD44-ko LM3 cells. WT, wild type; IB, immunoblot; WCE, whole-cell extract.

We performed verification experiments to dissect the underlying mechanism of HMMR regulating CD47 expression. We observed that overexpression of HMMR in LM3 and Huh7 cells led to increased phosphorylation of FAK (Tyr^397^) and SRC (Tyr^416^) accompanied by up-regulated CD47 expression (Fig. 4A and fig. S5A). While down-regulated HMMR expression decreased the phosphorylation of FAK (Tyr^397^) and SRC (Tyr^416^) and also accompanied by down-regulated CD47 expression (Fig. 4B and fig. S5B). We further introduced inhibitors to investigate the role of activated FAK and SRC in sustaining CD47 expression. Results showed that FAK inhibitor decreased the phosphorylation of FAK and SRC, accompanied by down-regulated CD47 expression in vector and OvHMMR LM3 and Huh7 cells (Fig. 4C and fig. S5C). CD44 is a cell surface adhesion receptor that interacts with HMMR to regulate the metastasis of cancer cells. To dissect the mechanism by which HMMR activates FAK/SRC, we pulled down HMMR from LM3 and Huh7 cell lysates using specific antibodies. We observed reciprocal coimmunoprecipitation of endogenous HMMR, CD44, FAK, and SRC, indicating that these proteins could form a complex (Fig. 4D and fig. S5D). Immunoprecipitates obtained with an anti-HMMR antibody in shHMMR cells showed notably decreased levels of coprecipitated SRC and FAK compared with shControl LM3 cells (Fig. 4E). Furthermore, immunoprecipitates obtained with an anti-CD44 antibody in shHMMR LM3 cells showed notably decreased levels of coprecipitated SRC, FAK, and HMMR compared with shControl LM3 cells (Fig. 4F). To investigate whether CD44 is an essential factor for HMMR to activate FAK/SRC and sustain CD47 expression. We constructed HMMR overexpressed liver cancer cells upon CD44 knockout (CD44-ko). Results showed that gain of HMMR expression could up-regulate CD47 expression by activating the phosphorylation of FAK and SRC independent of CD44 (Fig. 4G and fig. S5E). Intriguingly, we found that 62.9% of the patients with HMMR^high^ and CD44^low^ expression could sustain high CD47 expression (fig. S5F). Moreover, immunofluorescence staining showed that the colocalization of HMMR and CD44 was mainly found on the membrane of cancer cells (Fig. 4H). HMMR could also be detected in the cytoplasm and had a colocalization with FAK (Fig. 4H). To further demonstrate that HMMR could interact with FAK independent of CD44, we pulled down HMMR from CD44-ko LM3 cell lysates using specific antibodies. We observed reciprocal coimmunoprecipitation of HMMR, FAK, and SRC complex in CD44-ko cells, indicating that HMMR, FAK, and SRC could form a complex independent of CD44 (Fig. 4I). We used specific antibodies to pull down CD44 from the wild-type (WT) and CD44-ko LM3 cell lysates. The results demonstrated that the reciprocal coimmunoprecipitation of HMMR, FAK, CD44, and SRC existed in the WT cells while absent in the CD44-ko cells (Fig. 4J). These data together suggest that HMMR could activate the FAK/SRC signaling pathway independent of CD44 to sustain CD47 expression.

### HMMR interacts with FAK with the C-terminal in the cytoplasm

**Fig. 5.**
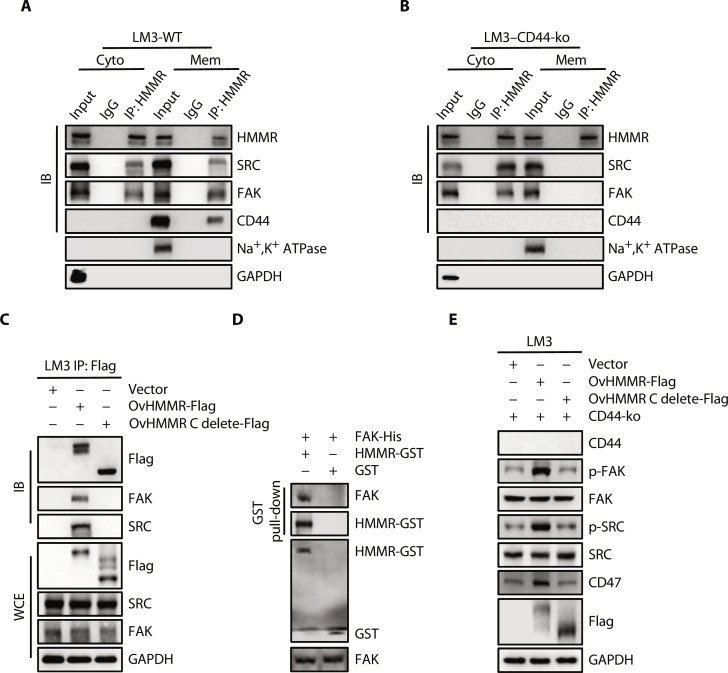
HMMR binds to FAK directly in the cytoplasm to sustain CD47 expression. (**A** and **B**) Immunoprecipitation analysis of the interaction between HMMR, FAK, SRC, and CD44 in cytoplasm and membrane separate from WT and CD44-ko LM3 cells. (**C**) Immunoprecipitation analysis of the interaction between HMMR, FAK, SRC in vector, HMMR-Flag, and HMMR C terminus delete-Flag LM3 cells. (**D**) GST pull-down analysis of the interaction between HMMR and FAK. HMMR-GST (2 μg) and FAK-His (2 μg) purified proteins were incubated in vitro overnight. (**E**) Immunoblotting analysis of indicated proteins in Vector, HMMR-Flag, and HMMR C terminus delete-Flag LM3 cells with CD44-ko. Na^+^, K^+^ ATPase, Na^+^- and K^+^-dependent ATPase; GAPDH, glyceraldehyde-3-phosphate dehydrogenase.

To determine whether endogenous HMMR could activate the phosphorylation of FAK (Tyr^397^) and SRC (Tyr^416^) independent of CD44. We separated the membrane and cytoplasmic component from LM3 and Huh7 cells. We pulled down HMMR from the cytoplasmic and membrane lysates using specific antibodies, separately. Results showed that HMMR-CD44-FAK-SRC could form a four-protein complex in the cell membrane component, and HMMR-FAK-SRC could form a three-protein complex in the cytoplasmic component (Fig. 5A and fig. S6A). We then pulled down HMMR from the cytoplasmic and membrane lysates using specific antibodies in the CD44-ko cells. Results showed that HMMR could form a complex with FAK and SRC in the cytoplasmic components while has nothing to do with the membrane components (Fig. 5B and fig. S6B). To further investigate whether there is a direct interaction between HMMR and FAK protein, we conducted domain-mapping experiments and found that the C-terminal region of HMMR is required for FAK interaction (Fig. 5C and fig. S6C). Glutathione S-transferase (GST) pull-down assay demonstrated that HMMR could interact directly with FAK in a cell-free condition (Fig. 5D). By overexpression of the full-length and truncated HMMR in CD44-ko Huh7 and LM3 cells, we found that overexpression of full-length HMMR could up-regulate the phosphorylation of FAK (Tyr^397^) and SRC (Tyr^416^). However, C-terminal truncated HMMR has no influence on the phosphorylation of FAK, SRC, and CD47 expression (Fig. 5E and fig. S6D). These results demonstrate that HMMR could interact directly with cytoplasmic FAK with the C-terminal region to sustain CD47 expression in HCC.

### The HMMR-FAK axis activates the NF-κB signaling pathway to sustain CD47 expression

**Fig. 6.**
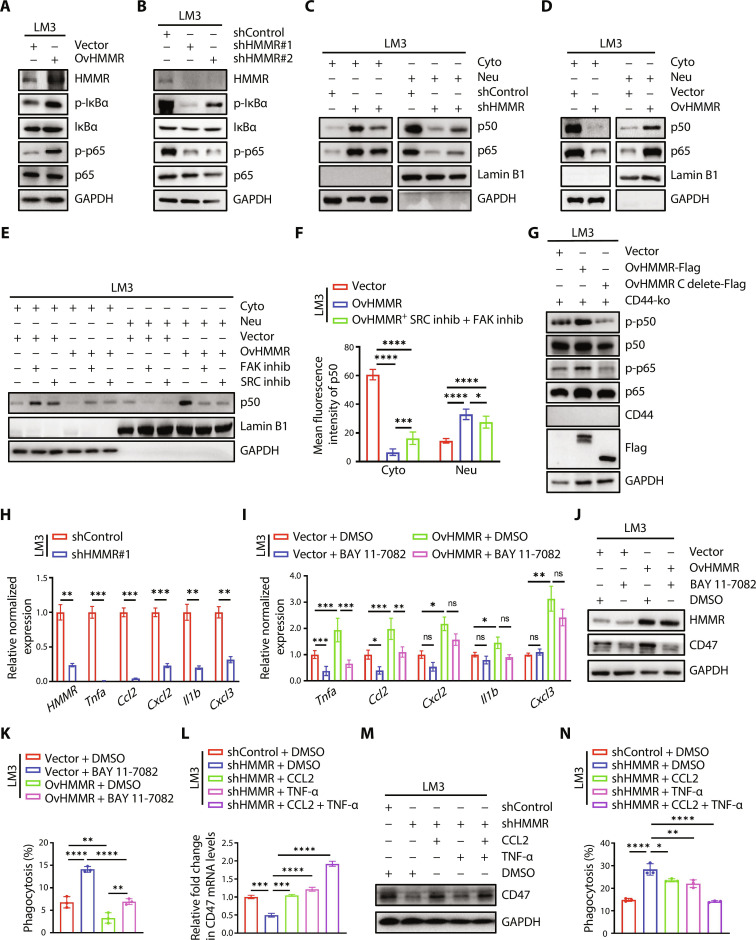
HMMR-FAK axis activates the NF-κB signaling pathway to sustain CD47 expression. (**A** and **B**) Immunoblotting analysis of indicated proteins in Vector and OvHMMR or in shControl and shHMMR LM3 cells. (**C** and **D**) Immunoblotting analysis of indicated proteins in the cytoplasm and nucleus separate from shControl and shHMMR or in Vector and OvHMMR LM3 cells. (**E**) Immunoblotting analysis of indicated proteins in the cytoplasm and nucleus separate from Vector and OvHMMR LM3 cells with or without FAK inhibitor (10 μM, 24 hours) and SRC inhibitor treatment (10 μM, 24 hours). (**F**) Statistics analysis of the nuclear translocation of p50 in Vector and OvHMMR LM3 cells with or without FAK and SRC inhibitor treatment. (**G**) Immunoblotting analysis of indicated proteins in Vector, HMMR-Flag, and HMMR C terminus delete-Flag in LM3-CD44-ko cells. (**H**) Quantitative real-time polymerase chain reaction (qRT-PCR) analysis of indicated genes in shControl and shHMMR LM3 cells. (**I**) qRT-PCR analysis of indicated genes in Vector and OvHMMR cells with or without BAY 11-7082 treatment. (**J**) Immunoblotting analysis of indicated proteins in Vector and OvHMMR LM3 cells with or without BAY 11-7082 treatment. (**K**) Flow cytometric analysis of phagocytosis in Vector and OvHMMR LM3 cells with or without BAY 11-7082 treatment. (**L** and **M**) qRT-PCR (L) and immunoblotting (M) analysis of CD47 expression in shControl and shHMMR LM3 cells with or without CCL2 (0.1 μg/ml) or tumor necrosis factor–α (TNF-α) treatment (30 ng/ml) for 24 hours. (**N**) Statistical analyses for the phagocytosis of GFP-labeled shControl and shHMMR LM3 cells with or without CCL2 (0.1 μg/ml) or TNF-α treatment (30 ng/ml). The data are presented as means ± SEM. The *P* values are calculated by unpaired, two-tailed Student’s *t* test, or two-way ANOVA. *P < 0.05, **P < 0.01, ***P < 0.001, and ****P < 0.0001. ns, nonsignificant.

We next investigated the downstream signaling of the HMMR-FAK axis. Western blot analysis showed that inhibitor kappa B alpha (p-IκBα) (Ser^32^) and p-p65 (Ser^536^) were markedly increased by OvHMMR HCC cells (Fig. 6A and fig. S7A), while phosphorylation of IκBα (Ser^32^) and p65 (Ser^536^) markedly decreased in shHMMR HCC cells (Fig. 6B and fig. S7B). We further analyzed the nuclear translocation of p50 and p65 in HMMR-overexpressed or down-regulated LM3 cells. Results showed that down-regulated HMMR expression decreased the nuclear translocation of p50 and p65 (Fig. 6C), while overexpression of HMMR increased the nuclear translocation of p50 and p65 (Fig. 6D). We then used FAK and SRC inhibitors to treat HCC cells, which notably decreased the nuclear translocation of p50 in vector and HMMR-overexpressed LM3 cells (Fig. 6E). We then examined the role of FAK/SRC signaling activation in HMMR-overexpressed liver cancer cells by immunofluorescence staining. Results showed that FAK and SRC inhibitors could decrease the nuclear translocation of p50 in HMMR-overexpressed liver cancer cells (Fig. 6F). We then conducted domain mapping and found that the C-terminal region of HMMR is required for the phosphorylation of p50 and p65 in HMMR-overexpressed Huh7 and LM3 cells (Fig. 6G and fig. S7C). Several downstream cytokines of the nuclear factor κB (NF-κB) signaling pathway, including *Ccl2* and *Tnfa*, were down-regulated in HMMR knockdown LM3 cells (Fig. 6H). Moreover, the up-regulated mRNA level of *Ccl2* and *Tnfa* in HMMR-overexpressed cells could be antagonized using an NF-κB signaling inhibitor, BAY 11-7082 (Fig. 6I). BAY 11-7082 could decrease CD47 expression in HMMR-overexpressed LM3 cells (Fig. 6J). Moreover, the inhibition of phagocytosis regulated by the overexpression of HMMR in LM3 cells could be reversed by BAY 11-7082 (Fig. 6K). We further revealed that adding CCL2 and tumor necrosis factor–α (TNF-α) could up-regulate CD47 expression accompanied by decreased phagocytosis efficiency (Fig. 6, L to N). These results indicate that HMMR-FAK/SRC–NF-κB signaling pathway could regulate the expression of CCL2 and TNF-α, accompanied by CD47 expression in HCC cells.

### HMMR knockdown enhances anti–PD-1 treatment to reduce liver cancer growth

**Fig. 7.**
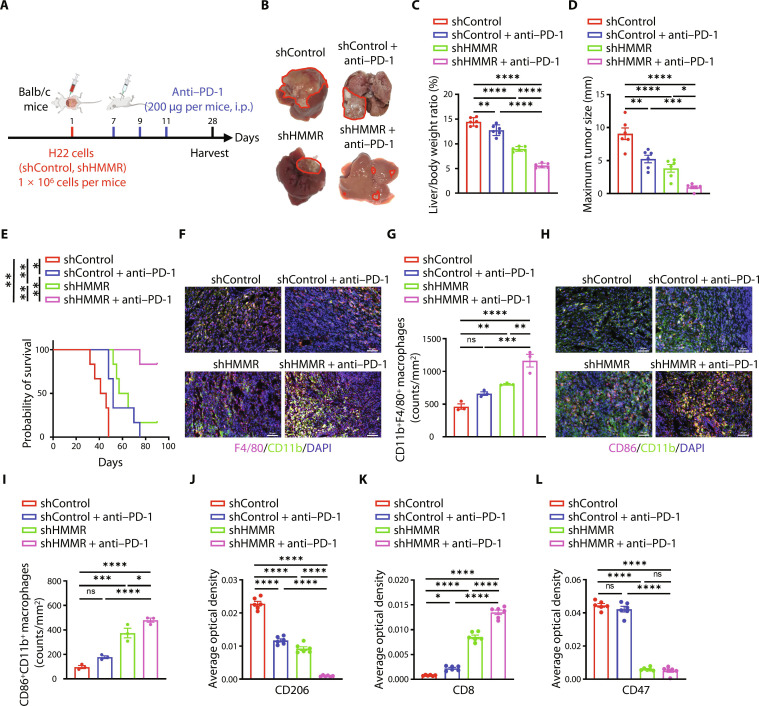
HMMR knockdown enhances anti–PD-1 treatment to reduce liver cancer growth. (**A**) Experimental schema for tumor implantation and antibody blockade treatment. Tumor cells were orthotopic injected into Balb/c mice. Seven days after tumor implantation, mice were treated with anti–PD-1 antibody (200 μg per mice, i.p.). Mice are euthanized in 28 days (*n* = 6). (**B**) Representative images of liver cancer at 28 days following liver orthotopic injection of H22 cells. (**C** to **E**) Bar plots of liver to body weight ratio (C), maximum tumor size (D), and prognosis of indicated groups (E). (**F** to **I**) Immunofluorescence staining and statistical analysis of F4/80^+^CD11b^+^ and CD86^+^CD11b^+^ macrophages of indicated groups. Scale bars, 50 μm. (**J** to **L**) Statistical analysis of CD206 (J), CD8 (K), and CD47 (L) expression by immunohistochemical staining in mouse liver cancer: Average optical density = integrated optical density (IOD)/area. All experiments were carried out at least in triplicate, and the data are presented as means ± SEM. The *P* values are calculated by log-rank test or two-way ANOVA. *P < 0.05, **P < 0.01, ***P < 0.001, and ****P < 0.0001. ns, nonsignificant.

Considering the dynamic interaction between macrophages and T cells in vivo, we wondered whether the enhanced infiltration of macrophages regulated by down-regulated HMMR could enhance the efficiency of immune checkpoint inhibitors. We conducted an animal experiment by using an in situ liver cancer mouse model. Results showed that HMMR knockdown could decrease tumor growth, and the combination of HMMR knockdown and anti–programmed cell death protein 1 (PD-1) treatment had the preferable therapeutic effect compared with HMMR knockdown or anti–PD-1 treatment alone (Fig. 7, A to E). Moreover, we evaluated the infiltration of CD11b^+^F4/80^+^ and CD86^+^CD11b^+^ macrophages in the indicated four groups. The results demonstrated that shHMMR increased the infiltration of CD11b^+^F4/80^+^ and CD86^+^CD11b^+^ M1 macrophages (Fig. 7, F to I). The immunohistochemical staining showed that shHMMR could decrease the expression of CD47, the inflitration of CD206^+^ macrophages, and enhance the intratumoral infiltration of CD8^+^ T cells (Fig. 7, J to L, and fig. S8A). Conclusively, these results demonstrate that targeting HMMR could enhance treatment efficiency of anti–PD-1 in liver cancer.

## DISCUSSION

TAMs are abundantly infiltrated in the immune microenvironment, which contribute to the metastasis, relapse, and drug resistance of HCC (*19*, *20*). Because of the high-degree plasticity of TAMs, important pathways regulating the infiltration and polarization of TAMs during tumor progression have been identified, which include inhibition of the recruitment of macrophages to tumors, repolarization of TAMs toward an antitumor phenotype, and other therapeutic strategies that elicit macrophage-mediated phagocytosis (*21*, *22*). In this study, we found with transgenic mice model that HMMR^−/−^ could decrease liver cancer growth by enhancing phagocytosis. Depletion of macrophages could reverse the decreased tumor growth in vivo. These findings indicate that HMMR is a potent antiphagocytic signal capable of directly protecting cancer cells from engulf by macrophages. This functional role of HMMR in immune escape expanded the original understanding of the pathophysiological function of including proliferation and metastasis of cancer cells (*11*).

Another interesting finding in our study is that HMMR could activate CD47 expression to enhance the immune escape. CD47 is a widely expressed transmembrane glycoprotein expressed in cancer cells, which is capable of inhibiting macrophage-mediated phagocytosis (*23*, *24*). Despite that antibodies designed for blocking CD47 have shown function of breaking the “don't eat me” signal, the use of CD47 monoclonal antibodies is limited due to the most common adverse events, including anemia and thrombocytopenia (*23*, *25*). HMMR interacting with FAK to activate downstream NF-κB signal is an important upstream regulatory mechanism for CD47 expression. Targeting HMMR to reduce CD47 expression on cancer cells and to stimulate macrophages to phagocytose tumor cells was found to be particularly crucial in mitigating systemic adverse reactions associated with the clinical use of blocking antibodies. Targeting HMMR offers a promising treatment strategy for patients with HCC. As per the previous report, the membrane-expressed HMMR could recruit FAK/SRC to form a complex in a CD44-dependent manner (*26*, *27*). Here, we provided evidence to demonstrate a noncanonical HMMR cytoplasmic regulatory signal in a CD44-independent manner that HMMR could recruit and activate FAK through the C-terminal region in the cytoplasm. This finding may explain why the patients with HMMR^high^CD44^low^ expression could also maintain a higher CD47 expression, which suggested that HMMR could be an indicator of immune escape and independent prognostic biomarker of patients with liver cancer.

HMMR is predicted to be a largely coiled-coil protein with microtubule binding domains at the N terminus and a bZip motif at the C terminus (*28*). The C-terminal bZip motif in HMMR is reported to bind in an ionic manner to hyaluronan and heparin. It is also known that the coiled-coil stalk and the N terminus of HMMR with two microtubule binding subdomains could also function to interact with downstream effectors such as CHICA/FAM83D (*29*). Whether HMMR interacts with CD47 through the other domains remains further investigations.

Various studies report that both the canonical and noncanonical NF-κB pathway are important regulators of inflammatory and cancer progression (*30*–*32*). Our study revealed that the cytoplasmic complex of HMMR and FAK could activate FAK/SRC signaling pathway and the downstream nuclear translocation of the p50/p65 heterodimer, which is essential for NF-κB signaling activation. The nuclear translocation of the p50/p65 heterodimer favored a pro-inflammatory environment and induced CCL2 and TNF-α production that helped sustain CD47 expression and facilitated immune escape. Except for the activated canonical NF-κB signaling, Janus kinase/signal transducers and activators of transcription 1 (STAT1) and interleukin-6 (IL-6)/STAT3 pathways and several downstream cytokines including interferon-γ (IFN-γ), TNF-α, and IL-1β were also reported to participate in the regulation of CD47 expression (*33*). FAK/SRC pathway is one of these pathways regulated by HMMR. It is also tempting to speculate that HMMR could interact with other molecules in the cytoplasm besides CD44, which needs to be further explored.

Immunotherapy using immune checkpoint inhibitors targeting the PD-1/PD-L1 pathway is the backbone of systemic therapies, which is only beneficial for partial patients in clinical practice (*34*–*36*). TAMs triggered inhibition of T cell–mediated antitumor immune response is the major contributor for treatment failure (*37*–*39*). In this study, we found that loss of HMMR could enhance the infiltration of CD8^+^ T cells, which suggested that HMMR could be used as a molecular target whose modulation could be synergistic with anti–PD-1 (*40*). In clinical practice, the patients with HMMR low expression might be more effective for anti–PD-1 treatment.

**Fig. 8.**
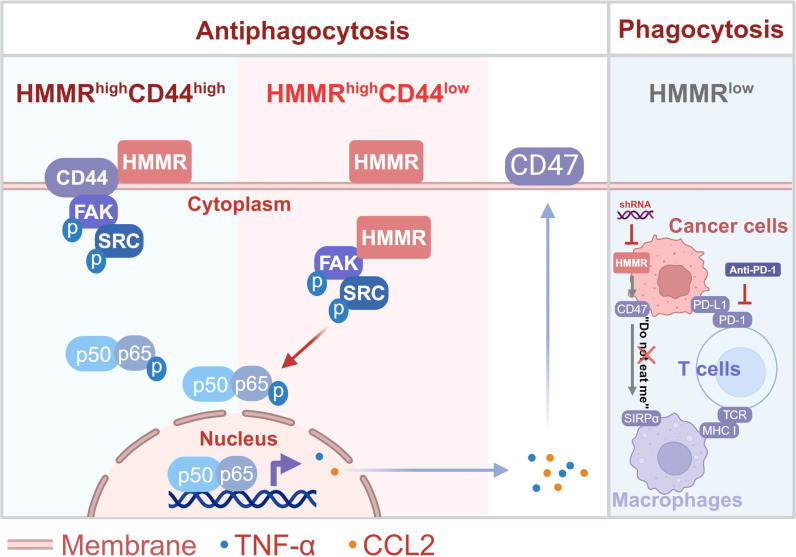
Schematic model. The schematic model shows that HMMR activates cytoplasmic FAK/SRC signaling independent of CD44 to trigger immune evasion of hepatocellular carcinoma by inactivating phagocyte killing.

In summary, our study identified that loss of HMMR putting the brakes of “don't eat me” signal. The cytoplasmic HMMR could recruit FAK/SRC to activate NF-κB signaling and sustain CD47 expression independent of CD44 (Fig. 8). Targeting HMMR provided a promising treatment strategy that could synergistically improve the treatment efficiency of immunotherapy. Our study also provided better prediction for the patients with HMMR low expression might have better treatment efficiency for anti–PD-1. These results provided another treatment strategy with particular promise for HCC treatment that antiphagocytosis immunotherapy independent of blocking CD47.

## MATERIALS AND METHODS

### Human samples

This study was approved by the Ethics Committee of Sichuan Province People’s Hospital, School of Medicine, University of Electronic Science and Technology of China (no. 2023182). Liver cancer samples were collected and analyzed following the acquisition of informed consent from all patients. The patients’ information was supplied in the tables S1 and S2.

### Cell culture

Human hepatoma cell lines Huh7 and LM3, murine hepatoma H22, and human embryonic kidney (HEK) 293T cells were obtained from the American Type Culture Collection (ATCC) and authenticated by the Cell Bank of Type Culture Collection of the Chinese Academy of Science, which were grown in Dulbecco’s modified Eagle’s medium (DMEM) or RPMI-1640 supplemented with 10% fetal bovine serum, penicillin (100 U/ml), and streptomycin (100 μg/ml). Cell cultures were maintained at 37°C and 5% CO_2_.

### Plasmids and shRNAs

Full-length human HMMR in the pLVX-CMV-EGFP-3FLAG-PGK-Puro vector and full-length human CD47 in the CMV-MCS-EGFP-SV40- Neomycin vector were purchased from GeneChem (Shanghai, China) for the overexpression assay. HMMR-truncated plasmids were built by Guangzhou Hanyi Biotech (Guangzhou, China). Lenti-CRISPR v2 was a gift from Feng Zhang (Addgene, plasmid #52961; http://n2t.net/ addgene:52961; RRID: Addgene_52961). The single-guide RNA (sgRNA) was shown below: CD44 sgRNA-1 (5′-TCGCTACAGCATCTCTCGGA-3′) and CD44 sgRNA-2: (5′-ACATATTGCTTCAATGCTTC-3′). Short hairpin RNA (shRNA) specific for targeting HMMR and CD47 were obtained from GeneChem (Shanghai, China): HMMR shRNA-1 (5′-GCCAACTCAAATCGGAAGT AT-3′), HMMR shRNA-2 (5′-TCACTTGGTCCTACCTATTAT-3′), and CD47 shRNA (5′-GCCTTGGTTTAATTGTGACTT-3′). Human HEK293T cells (American Type Culture Collection) were cultured in six-well plates until ~85% confluent and cotransfected with 2 μg of target plasmids, 1 μg of pMD2.G, and 1 μg of psPAX2 lentivirus packaging vectors using Lipofectamine 2000 (Invitrogen) according to the manufacturer’s protocol. The supernatants with the virus were harvested twice at 48 and 72 hours, filtered through a 0.45-μm syringe filter, and frozen in liquid nitrogen.

### Quantitative real-time PCR

Total RNA was extracted from cancer cells using Trizol Reagent (Invitrogen, USA). Quantitative real-time polymerase chain reaction (qRT-PCR) was performed using the SYBR Prime Script RT-PCR Kit (TaKaRa, Japan) on a CFX96 Touch Real-time Detection System (Bio-Rad, USA). Primers of cancer genes and the product sizes were listed in table S3. Glyceraldehyde-3-phosphate dehydrogenase was amplified as an internal control. Each sample was tested at least in triplicates.

### Multiplex immunofluorescence staining (multiplex IF)

Multiplex immunofluorescence for anti-CD163 (1:150; Cell Signaling Technology, catalog no. 93498S), anti-CD8α (1:200; Cell Signaling Technology, catalog no. 85336S), anti-CD11b (1:200; Bioss, catalog no. bs-1014R ), anti-F4/80 (1:200; Abcam,catalog no. ab6640), and anti-CD86 (1:200; Cell Signaling Technology, catalog no. E5W6H) were performed using 5-μm sections of formalin-fixed paraffin-embedded tumor samples by sequential staining after antigen retrieval in cell conditioning solution (pH 8.5) in a water bath at 98°C for 30 min. The Opal Polymer HRP Ms + Rb was used for the primary antibody detection and Opal 6-Color Manual IHC (Akoya Biosciences, catalog no. NEL811001KT), with four reactive fluorophores (Opal 620, Opal 540, Opal 520, and Opal 570) plus 4′,6-diamidino-2-phenylindole (DAPI) nuclear counterstain, were added according to the manufacturer’s instructions. The slides were imaged using Vectra 3.0 spectral imaging system (PerkinElmer) according to previously published instructions.

### Immunofluorescence staining

For immunofluorescence staining, tumor cells were prepared. Tumor cells were blocked with pre-immune goat serum at 37°C for 30 min and then incubated with primary antibodies at 4°C overnight. Antibody information used in the experiments will be provided in table S4. The tumor cells were subsequently washed in phosphate-buffered saline (PBS) and incubated at 37°C for 1 hour with Cy3- or Cy5-conjugated goat anti-rabbit or anti-mouse IgG antibodies (1:1000; Invitrogen, USA). Nuclei were counterstained with Hochest 33258. Cells were observed under laser confocal scanning microscopy (Leica TCS-SP5, Germany). In addition, tumor tissues from human surgical biopsy specimens obtained from 14 patients with liver cancer were used in immunofluorescence staining to detect HMMR and CD47 protein expression. Cells were observed under a Nikon Eclipse Ti-S fluorescence microscope (Nikon, Tokyo, Japan).

### Immunohistochemistry

Tissues were fixed in 4% paraformaldehyde overnight, embedded in paraffin, and cut into sections at 5 μm in thickness. For the immunohistochemistry assay, the sections were incubated with primary antibodies at 4°C overnight after deparaffinization and antigen retrieval. Then, the sections were incubated with horseradish peroxidase–conjugated secondary antibodies for 20 min at 37°C. The nucleus was stained with hematoxylin. For quantification, Image-Pro Plus was used to analyze the optical density of the images. The average optical density, namely, integrated optical density/area, was calculated.

### Immunoblotting and immunoprecipitation

Cell lysates of Huh7 and LM3 liver cancer cells and tumor tissues were prepared using radioimmunoprecipitation assay buffer with protease inhibitors. Proteins were loaded onto a 10% SDS–polyacrylamide gel (SDS-PAGE) and transferred onto polyvinylidene difluoride (PVDF) membranes (Millipore, USA). Membranes were incubated with tris-buffered saline (TBS) blocking buffer containing 5% milk and subsequently with primary antibodies. The information on primary and secondary antibodies was provided in table S4. For immunoprecipitation, cell lysates were prepared and incubated with antibodies overnight, followed by incubation with 40 μl of Protein A/G agarose beads for 4 hours at 4°C. The beads were then washed four times with lysis buffer and subjected to immunoblotting of specific antibodies.

### Flow cytometry analysis

Tumor tissues were chopped, digested, and filtered through a 70-μm cell strainer to generate a single-cell suspension. Cells were incubated with red blood cell lysis for 15 min at 4°C and washed in PBS twice. Cell surface molecule staining was performed at 4°C for 30 min in PBS in the dark. The antibodies used in this study were provided in table S4. DAPI was used to exclude dead cells. Flow cytometry was performed on a FACS Calibur (BD Biosciences, USA). The cells were then analyzed, and results were calculated using FlowJo software (BD Biosciences, USA). Gating strategy for multicolor flow cytometry in DEN-induced liver cancer was shown in fig. S9A.

### Preparation of HMMR-GST and FAK (35-684)–His protein

Using pGEX-4T-1-HMMR-GST plasmid as a template, HMMR-GST protein was induced by 0.2 mM isopropyl-β-d-thiogalactopyranoside (IPTG) at 15°C overnight in BL21 (DE3). FAK (35-684)–His protein used pCZN1 as a vector and induced by 0.2 mM IPTG at 15°C overnight in Arctic-Express. The protein purification was performed by Zoonbio Biotechnology. The expression of HMMR-GST and FAK (35-684)–His protein was confirmed by Western blot analysis with anti-GST and anti-His antibodies, respectively.

### GST pull-down

GST pull-down assay was performed according to the Pierce GST Protein Interaction Pull-Down Kit (Thermo Fisher Scientific, #21516) instructions. In short, 200 μg of HMMR-GST or GST control protein were incubated with Glutathione Agarose in Tris Buffered Saline with Tween 20 (TBST) buffer at 4°C. After 1 hour of incubation, FAK-His protein was added and thoroughly mixed in a vibrating mixer at 4°C for 1 hour. Immunoprecipitates were then washed four times and analyzed by SDS-PAGE.

### Mice alleles

All mice experiments were conducted according to the experimental procedures approved by the Institutional Animal Care and Use Committee of Sichuan Province People’s Hospital. HMMR knockout (HMMR^−/−^) mice (strain no. T015690) were purchased from GemPharmatech (Nanjing, China). According to the structure of the HMMR gene, exon4-exon7 of the HMMR-201 (ENSMUST00000020579.8) transcript is recommended as the knockout region. In this project, we used CRISPR-Cas9 technology to modify the HMMR gene. Mouse genomic DNA was prepared for PCR genotyping through incubation of each sample in 200 μl of 0.05 M NaOH for 20 min at 98°C followed by the addition of 20 μl of 1 M tris-HCl (pH 7.5) at room temperature. Primers used in the PCR genotyping are as follows: for HMMR^Δexon4-7^ (5′-CTGTAG GTTGT GCTCCATCTCCG-3′ and 5′-TGAGGTCGAGGCC AGTTTGGTT-3′) [a 587–base pair (bp) product] and for HMMR wild-type (5′-GCTGGTGCTGGCTGATTGTTATG-3′ and 5′-AGGAAATGCCATGACCAGAAGC-3′) (a 353-bp product). Mice were maintained at temperatures and humidity ranges of 20° to 26°C and 30 to 70%, respectively. Mice were housed in standard cages under 12-hour light/12-hour dark cycles with ad libitum to access water and food and were randomly assigned to different experimental groups.

### Phagocytosis assay in vitro

BMDMs were extracted from the mouse tibia and femur and immediately plated in petri dishes with DMEM supplemented 20% L-929 conditioned medium. Cells were fed on day 2 and day 5. Seven days later, cells were stimulated by lipopolysaccharide (240 ng/ml) and IFN-γ (20 ng/ml) for 48 hours to induce M1-type macrophages. The induced M1 macrophages were cultured in serum-free DMEM for 12 hours and trypsinized. A total of 1 × 10^5^ M1 macrophages and an equal number of target cancer cells were added into the fluorescence-activated cell sorting tube in a total volume of 200 μl. All tubes were incubated at 37°C for 3 hours. After co-incubation, cells were incubated for 30 min with rat anti-mouse F4/80 antibody to stain macrophages. Cells were washed three times with serum-free DMEM in 200 μl of serum-free DMEM. Phagocytosis was assessed by flow cytometry (BD FACS). At least 1 × 10^4^ macrophages were counted per tube. For the microscopy-based assay, M1 macrophages were labeled with PKH26 (Sigma-Aldrich, USA) according to the manufacturer’s protocol, and 2 × 10^4^ macrophages were seeded overnight in a 24-well tissue culture plate. The next day, an equal amount of target cancer cells was spread to the plate and co-incubated with macrophages at 37°C. Three hours later, macrophages were extensively washed and imaged with a Fluorescence microscope (Nikon Eclipse Ti-S fluorescence microscope). The phagocytosis efficiency was calculated as the number of macrophages containing GFP^+^ cancer cells per 100 macrophages (*41*). The gating strategy for phagocytosis in vitro was shown in fig. S10A.

### In vivo phagocytosis assays

LM3-Vector&shControl, OvHMMR&shControl, Vector&shCD47, and OvHMMR&shCD47 were inoculated subcutaneously (1 × 10^6^ cells per mouse, *n* = 5 mice per group) of 6-week-old male Balb/c nude mice. LM3-shControl&Vector, shHMMR&Vector, shControl&Ov-CD47, and shHMMR&Ov-CD47 cells were inoculated subcutaneously (1 × 10^6^ cells per mouse, *n* = 4 mice per group) of 6-week-old male Balb/c nude mice. Sample size estimation of the experimental animals was based on the resource equation approach. Mice were monitored every week for the appearance of subcutaneous tumors. Twenty-one days later, mice were euthanized, tumor xenografts were isolated, and tumor volume (TV) and tumor weight were measured. TV was calculated using the following formula: TV (mm^3^) = *d*^2^ × *D*/2, where *d* and *D* represent the shortest and the longest diameters, respectively. Tumor tissues were harvested in 1× PBS, minced, and digested into a single-cell suspension in a mixed solution containing collagenase types 1 and 4 (1.5 mg/ml) in DMEM shaken at 120 rpm for 1 hour at 37°C. The suspension was filtered through a 70-μm cell strainer, and cells were washed three times with DMEM and resuspended in a cold flow buffer. Single-cell suspensions were incubated with rat anti-mouse CD11b and rat anti-mouse F4/80 at 4°C for 30 min, washed, and resuspended in cold flow buffer. Flow cytometric data were obtained using a FACS Calibur (BD Biosciences, USA) and analyzed with FlowJo software. The gating strategy for phagocytosis in vitro was shown in fig. S10B.

### Depletion of macrophages

Mice were intraperitoneally injected with clodronate liposomal or control liposomal (200 μl per mice; Yeasen, Shanghai, *n* = 4 per group) as the products specification described. The effect of macrophage depletion was analyzed by flow cytometry analysis.

### Anti–PD-1 treatment mice model

Balb/c mice were anesthetized and exposed the liver in sterile condition, and 1 × 10^6^ H22 cells with or without HMMR knockdown were orthotopically injected into the mouse liver by using microliter syringes. Anti–PD-1 treatment was performed every 2 days for three times at 200 μg per mouse. After 28 days, the mice were etuhanized, and the weight of the mice and liver were recorded. Mice livers were soaked in 4% paraformaldehyde for subsequent immunohistochemical experiments.

### Single-cell data integration and analysis

The scRNA-seq data have been downloaded from the Gene Expression Omnibus (GEO) database with accession number GSE149614. The dataset contains 21 samples from 10 patients. Normalization, dimensionality reduction, and clustering were accomplished with the Seurat 4.2.3 R package (*42*). After excluding normal tissue cells, the clustering results of all 29,630 cells were visualized using UMAP scatter plot and annotated by the singleR package. HCCs were annotated on the basis of the expression of GPC3, Midkine (MDK), epithelial cell adhesion molecule (EpCAM), and CD24 (*43*–*45*) and divided into HMMR^+^ HCC and HMMR^−^ HCC based on the expression of HMMR. Macrophages were annotated based on the expression of CD80, CD86, CD163, and MRC1 and divided into M1 macrophages and M2 macrophages based on the expression of the marker genes.

### Cell-cell interaction network analysis and coexpression between HMMR and CD47

The cell-cell interaction network was analyzed by the CellChat R package (version 1.1.2) (*46*), a tool specifically designed for studying ligand-receptor interactions in specific signaling pathways. To explore the relationship of HMMR^+^ HCC with immune cells, the gene expression matrix and metadata with principal cell annotations from cancer samples were used as input for the CellChat analyze. Essentially, the interactions between HMMR and the immune microenvironment were measured by quantifying the ligand-receptor pairs between HMMR^+^ HCC and immune cells, especially macrophages. Moreover, HCC was isolated and the coexpression relationship between HMMR and CD47 was examined. The results were visualized by UMAP and heatmap, and the coexpression relationship between HMMR and CD47 was tested by Pearson correlation method (*47*).

### Data collection and analysis

Liver cancer tissue sequencing data were downloaded from The Cancer Genome Atlas (TCGA) (https://portal.gdc.cancer.gov/) and GEO (www.ncbi.nlm.nih.gov/geo/) databases with accession number GSE14520. The TCGA dataset included sequencing data from 371 liver cancer samples and 50 normal liver tissue samples, along with survival status and overall survival data for the 371 patients with liver cancer. The GSE14520 dataset comprised 225 liver cancer samples and 220 normal liver tissue samples. We compared the differences in HMMR expression between HCC tissues and adjacent normal tissues in both TCGA and GSE14520 datasets. Survival curves were plotted after dividing patients from the TCGA cohort into high and low expression of HMMR and CD47 by res.cut function from the survival package. The log-rank function assessed survival differences among the patient groups. In our cohort analysis, we used the ggalluvial package to generate Sankey diagrams to display relationship between the protein expression of CD44 and CD47 in patients with highly expressed HMMR by immunohistochemical staining.
