# Supplementary material for: HMMR triggers immune evasion of hepatocellular carcinoma by inactivation of phagocyte killing
Source: Sci Adv. 2024 Jun 5;10(23):eadl6083. doi: 10.1126/sciadv.adl6083 (PMC11152120; doi:10.1126/sciadv.adl6083)

Science Advances
AAAS

Supplementary Materials for

HMMR triggers immune evasion of hepatocellular carcinoma by inactivation of phagocyte killing

Hong Wu *et al.*

Corresponding author: Chuan Xu, xuchuan100@163.com; Xiu-Wu Bian, bianxiuwu@263.net

Sci. Adv. **10**, eadl6083 (2024)
DOI: 10.1126/sciadv.adl6083

This PDF file includes:

Figs. S1 to S10
Tables S1 to S4

Fig. S1.

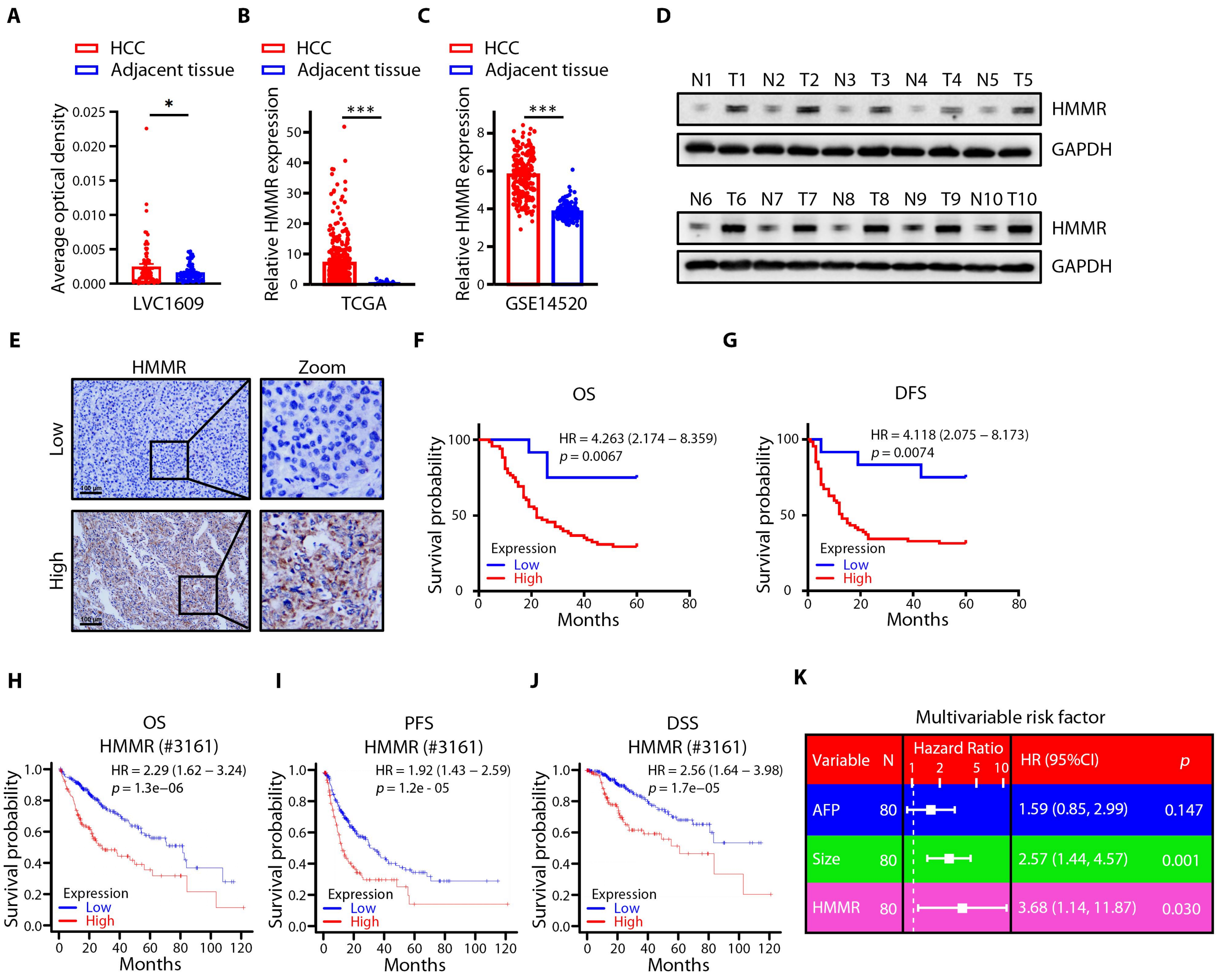

Fig. S1. High HMMR expression is correlated with the poor prognosis of HCC patients.

(**A**) Relative expression of HMMR in HCC tumor tissues and adjacent normal tissues by IHC staining (n = 80), Average optical density = IOD/area. (**B** and **C**) HMMR expression level in HCC tumor tissues and adjacent normal tissues of TCGA and GSE14520 databases. (**D**) The protein expression of HMMR in HCC tumor tissues and paired adjacent normal tissues by western blot. (**E**) Representative images show the expression of HMMR by IHC staining. Scale bars = 100 μm. (**F** and **G**) Kaplan–Meier analysis shows the overall survival (OS) (**F**) and disease-free survival (DFS) (**G**) between HMMR low and high expression in HCC patients (n = 80), cutoff value = 0.00027. (**H** to **J**) Kaplan–Meier analysis shows the overall survival (OS) (cutoff value = 277) (H), Progression-free survival (PFS) (cutoff value = 277) (I) and disease-specific survival rates (DSS) (cutoff value = 273) (J) with HMMR low and high expression in HCC patients. (**K**) Multivariate analysis of factors associated with OS of patients with HCC in the LVC1609 cohort. Data are presented as the mean ± SEM. The P values are calculated by unpaired, two-tailed Student's t test or log-rank test for survival. *$P < 0.05$; ***$P < 0.001$.

Fig. S2.

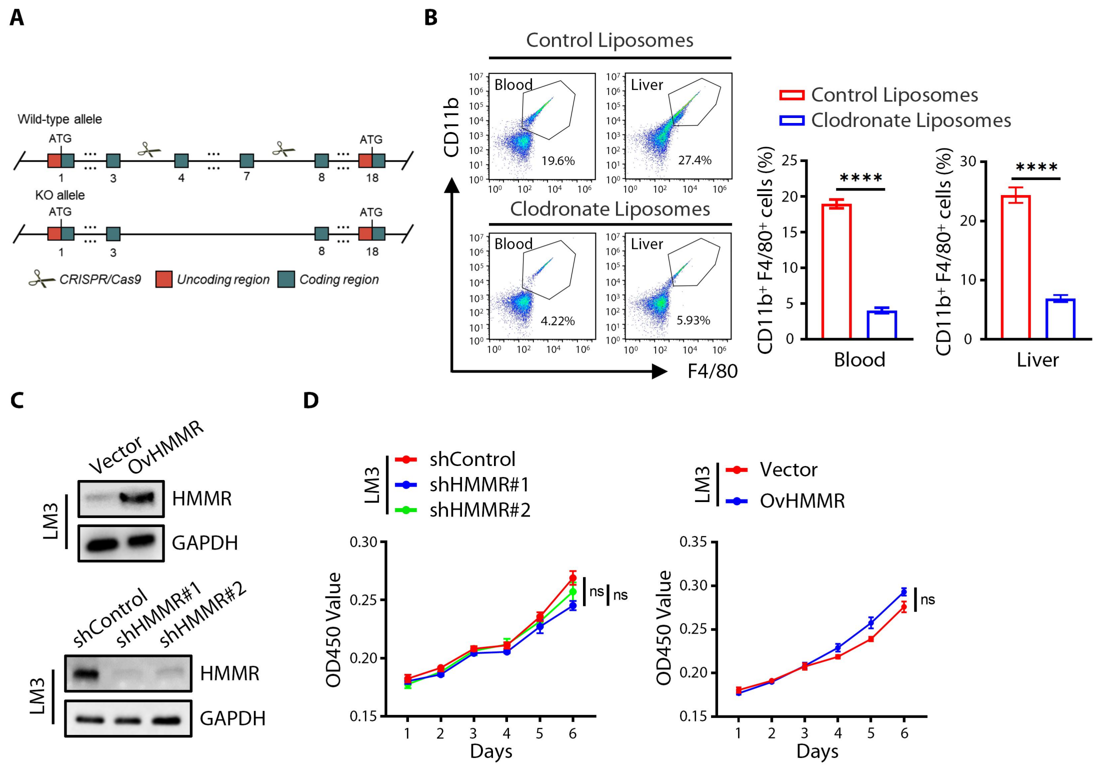

Fig. S2. HMMR has no influence on tumor growth *in vitro*.

(**A**) Using CRISPR/Cas9 technology to edit the HMMR gene and construct HMMR$^{-/-}$ mice model. (**B**) Flow cytometry analysis the proportion of macrophages percentage in the blood and liver tissues after clodronate-liposome treatment. (**C**) Immunoblotting analysis of indicated proteins in LM3 cells. GAPDH as internal control. (**D**) CCK8 assays of shControl and shHMMR LM3 cells or vector and OvHMMR LM3 cells. The *P* values are calculated by paired or unpaired, two-tailed Student's *t* test. ns: non-significant.

Fig. S3.

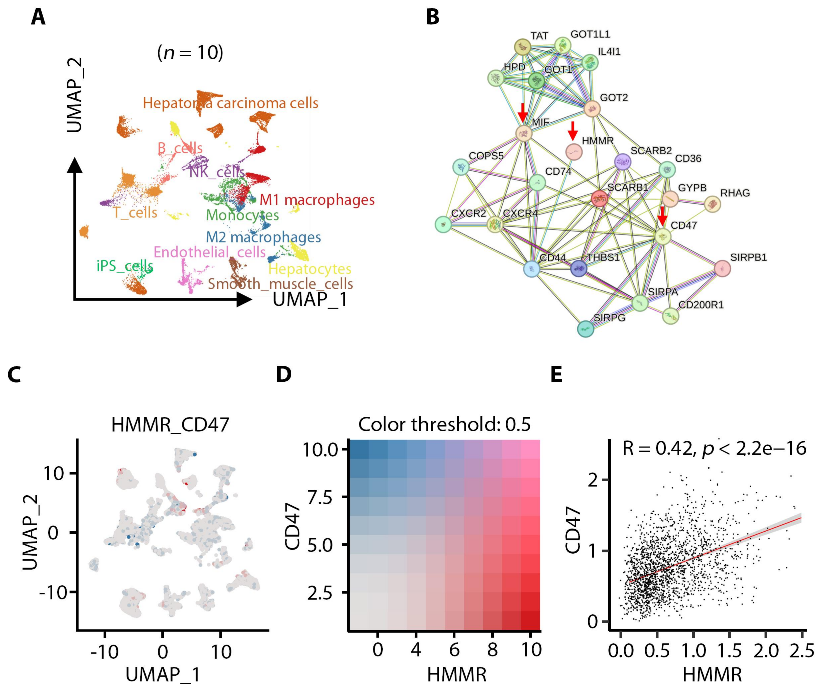

Fig. S3. HMMR expression correlated with CD47 expression.

(**A**) Uniform Manifold Approximation and Projection (UMAP) plot, showing the annotation and color codes for cell types in liver cancer patients (*n* = 10). The scRNAseq data was accessed from the GEO database (GSE149614). (**B**) Gene-gene interaction network. (**C** and **D**) UMAP plots show the co-expression of HMMR and CD47 across individual single HCCs. The co-expression matrix across HCCs with different expressions of HMMR and CD47. Red/blue dots represent HCCs expressing HMMR only/CD47 only, respectively. (**E**) The correlation (Pearson) between HMMR and CD47 across HCCs.

Fig. S4.

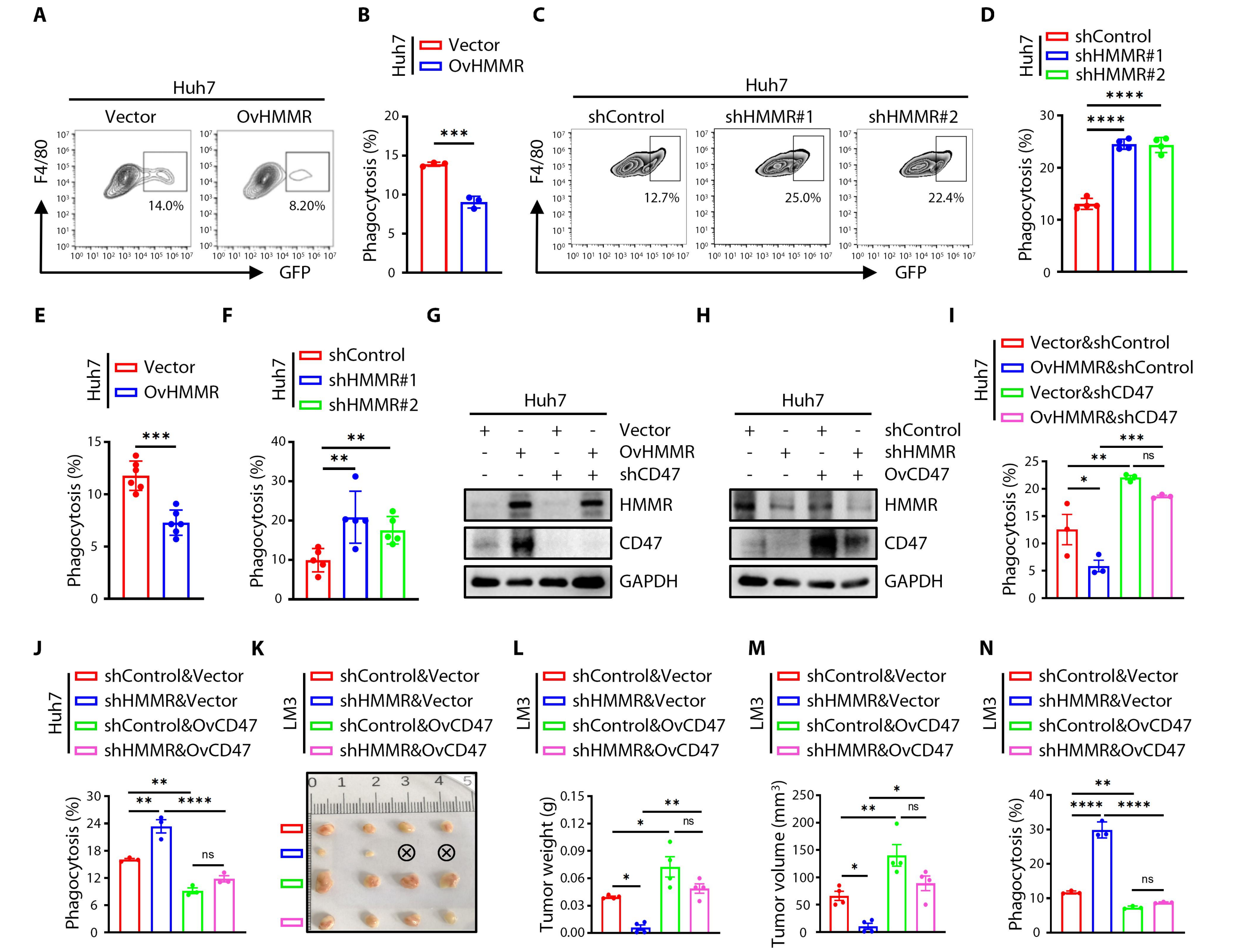

Fig. S4. HMMR expression influences the phagocytosis efficiency of tumor cells.

(**A** and **B**) Phagocytosis and statistical analysis of Vector and OvHMMR Huh7 cells by flow cytometric analysis. (**C** and **D**) Phagocytosis and statistical analysis of shControl and shHMMR Huh7 cells by flow cytometric analysis. (**E** and **F**) Phagocytosis assay of GFP-labeled indicated Huh7 cells and PKH26-labeled BMDMs assessed by confocal microscopy. (**G**) Immunoblotting analysis of indicated proteins in vector and OvHMMR Huh7 cells with or without CD47 knockdown. (**H**) Immunoblotting analysis of indicated proteins in shControl and shHMMR Huh7 cells with or without CD47 overexpression. (**I**) Statistical analysis the phagocytosis of vector and OvHMMR (GFP-labeled) Huh7 cells with or without shCD47 by F4/80 labeled BMDMs using flow cytometry. (**J**) Statistical analysis the phagocytosis of shControl and shHMMR (GFP-labeled) Huh7 cells with or without OvCD47 by F4/80 labeled BMDMs using flow cytometry. (**K** to **M**) Tumor image (K), Tumor weight (L), and tumor volume (M) of Balb/c nude mice inoculated with shControl and shHMMR LM3 cells with or without CD47 overexpression (1×10^6 inoculated cells/mice, $n = 5$ mice per group). (**N**) Phagocytosis of indicated LM3 xenografts is represented by the percentage of $GFP^+F4/80^+$ cells in total $F4/80^+$ cells. All experiments are carried out at least in triplicate and the data are presented as the mean ± SEM. The P values are calculated by unpaired, two-tailed Student's t test and two-way ANOVA. *$P < 0.05$; **$P < 0.01$; ***$P < 0.001$; ****$P < 0.0001$; ns: non-significant.

Fig. S5.

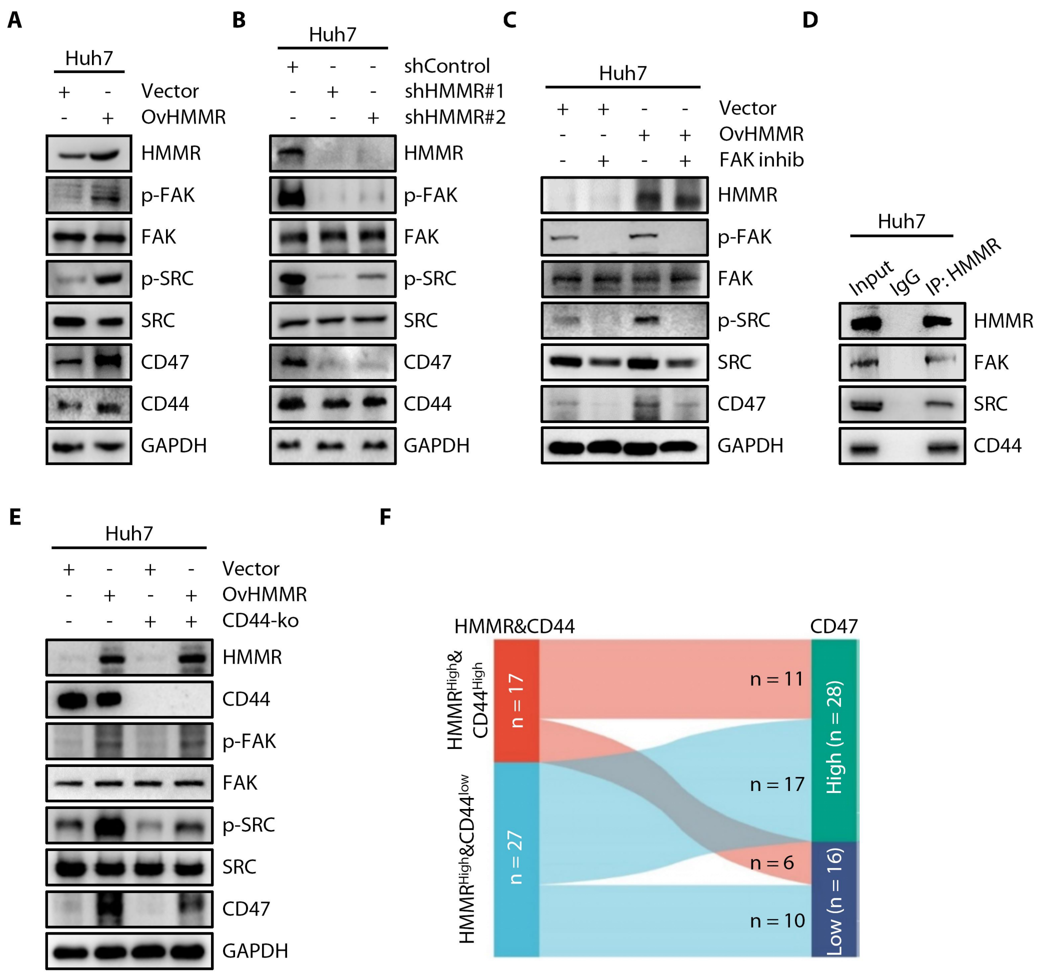

Fig. S5. HMMR activates FAK/SRC signaling independent of CD44 to sustain CD47 expression in HCC patients.

(**A**) Immunoblotting analysis of indicated proteins in vector and OvHMMR Huh7 cells. (**B**) Immunoblotting analysis of indicated proteins in shControl and shHMMR Huh7 cells. (**C**) Immunoblotting analysis of indicated proteins in vector and OvHMMR Huh7 cells with or without FAK inhibitor (10 uM) treatment for 24 hrs. (**D**) Immunoprecipitation analysis of the interaction between HMMR, FAK, SRC and CD44 in Huh7 cells. (**E**) Immunoblotting analysis of indicated proteins in vector and OvHMMR Huh7 cells with or without CD44 knockout. (**F**) Sankey diagram show the relationship between the protein expression of CD44 and CD47 in the HMMR^{high} cohort (n = 44) containing HMMR^{high}&CD44^{high} patients (n = 17), HMMR^{high}&CD44^{low} patients (n = 27).

Fig. S6.

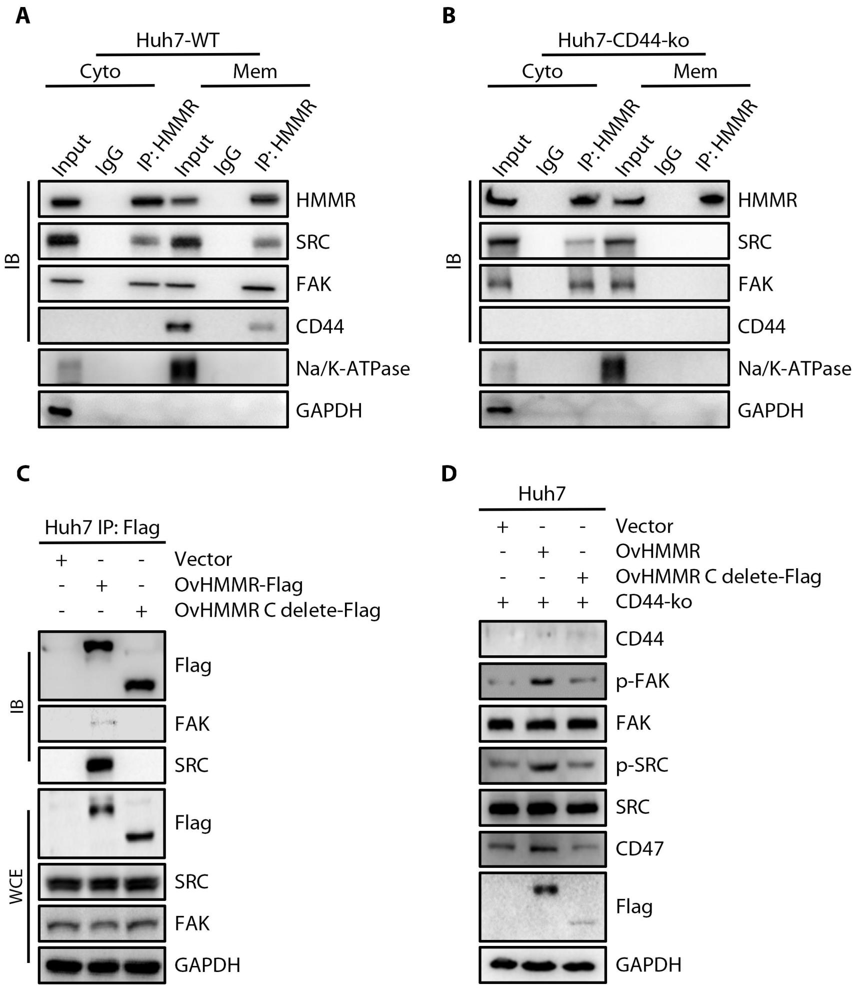

Fig. S6. HMMR binds to FAK directly in the cytoplasm to sustain CD47 expression.

(**A** and **B**) Immunoprecipitation analysis of the interaction between HMMR, FAK, SRC and CD44 in cytoplasm and membrane separate from WT and CD44-knockout Huh7 cells. IB, immunoblot. (**C**) Immunoprecipitation analysis of the interaction between HMMR, FAK, SRC in vector, HMMR-Flag, and HMMR C terminus delete-Flag Huh7 cells. (**D**) Immunoblotting analysis of indicated proteins in vector, HMMR-Flag, and HMMR C terminus delete-Flag Huh7 cells. WT, wild type; IB, immunoblot; WCE, whole-cell extract

Fig. S7.

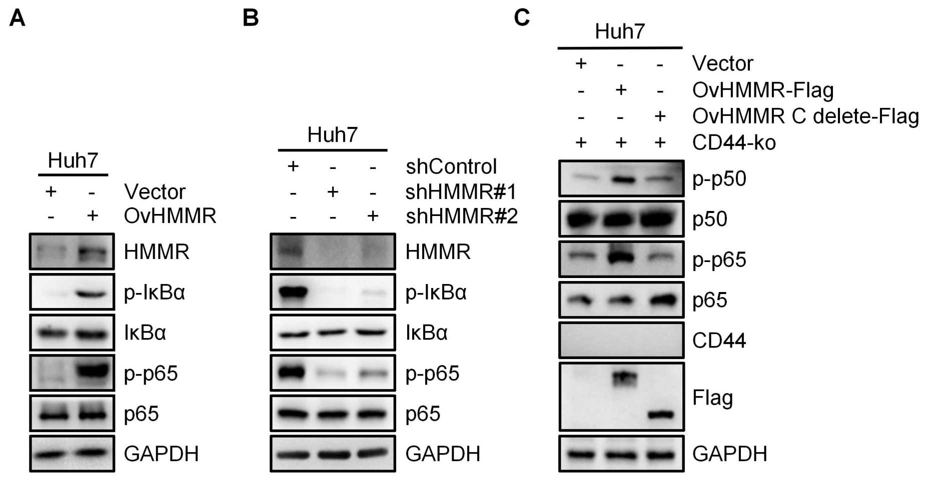

Fig. S7. HMMR activates NF-κB signaling pathway to sustain CD47 expression.

(**A**) Immunoblotting analysis of indicated proteins in Vector and OvHMMR Huh7 cells. (**B**) Immunoblotting analysis of indicated proteins in shControl and shHMMR Huh7 cells. (**C**) Immunoblotting analysis of indicated proteins in vector, HMMR-Flag, and HMMR C terminus delete-Flag Huh7 cells with CD44 knockout.

Fig. S8.

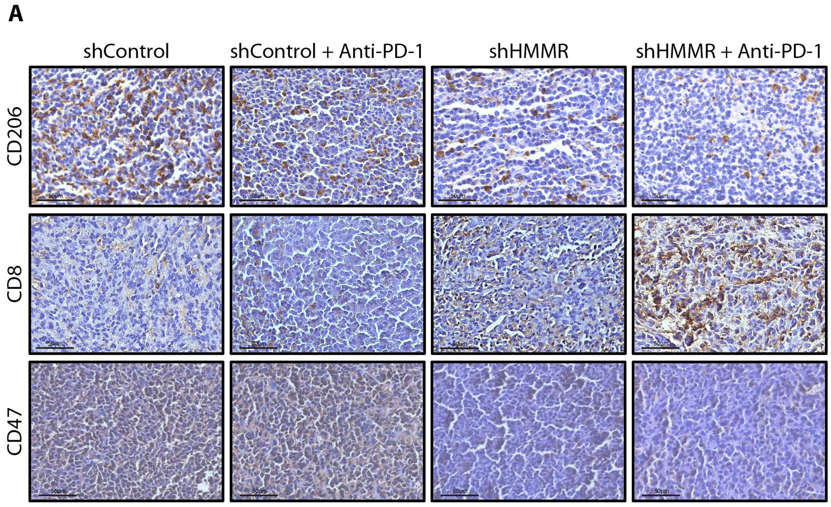

Fig. S8. Representative images of immunohistochemical staining.

(A) Representative images show immunohistochemical staining of CD206, CD8, and CD47 expression in indicated groups.

Fig. S9.

A

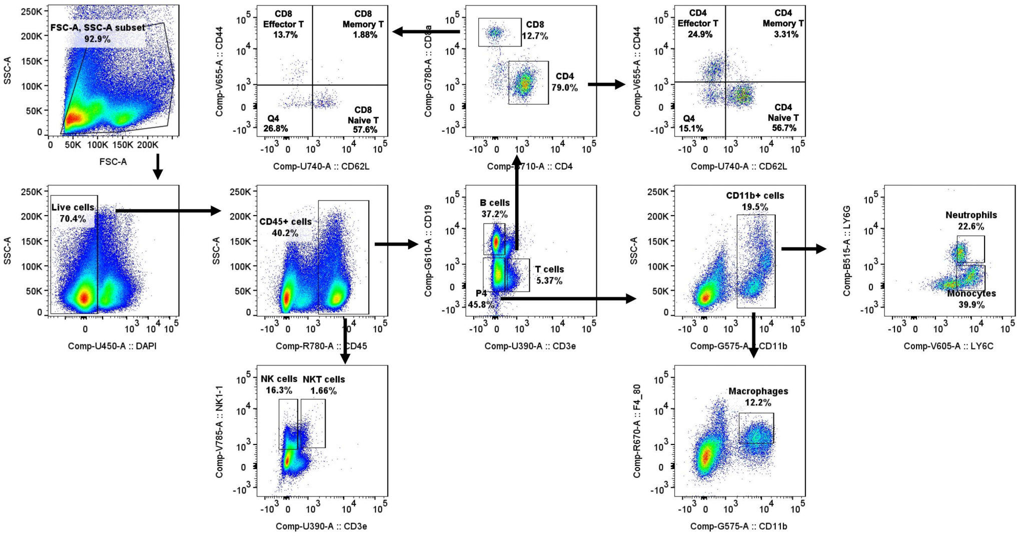

Fig. S9. Tumor-infiltrating immune cell multi-color flow cytometry detection.

(**A**) Gating strategy for multi-color flow cytometry.

Fig. S10.

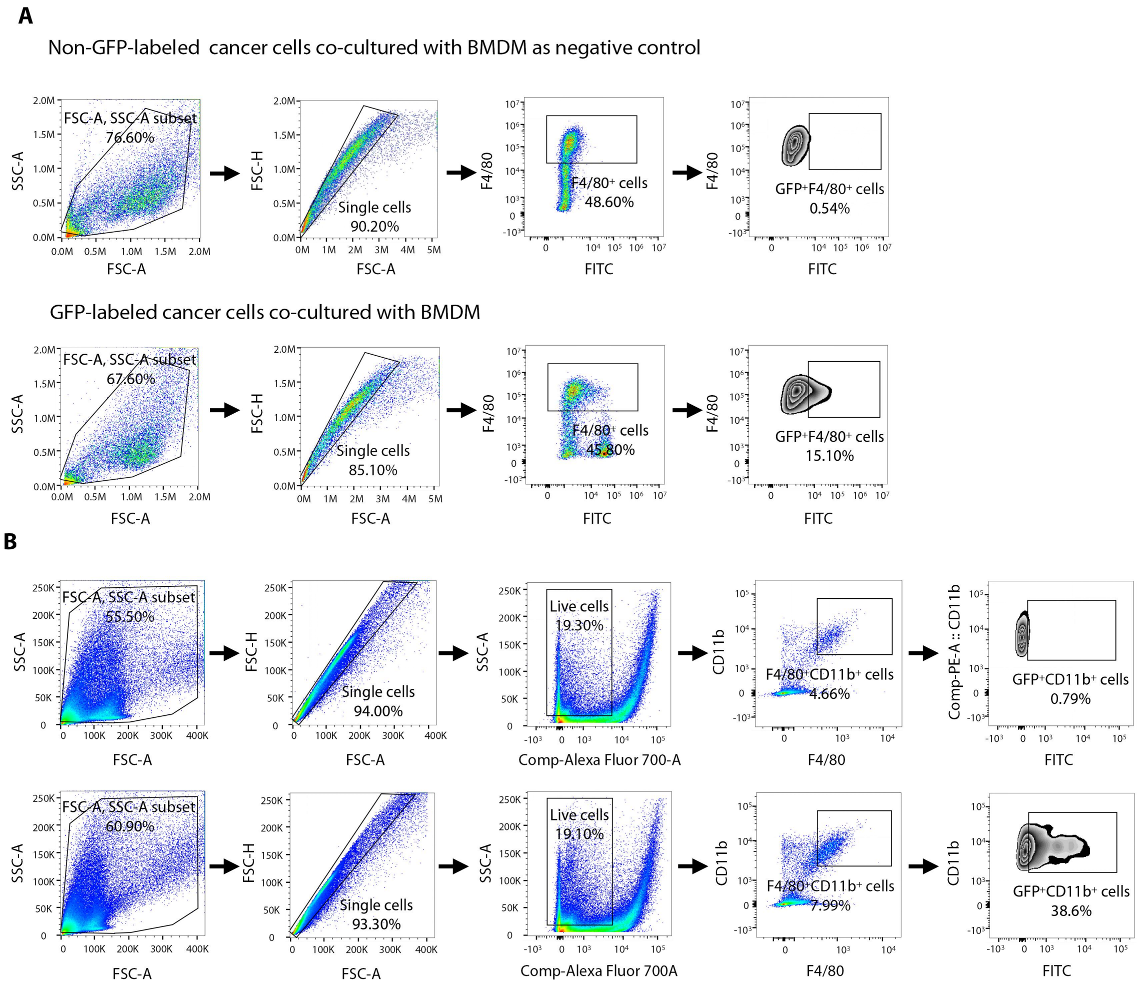

Fig. S10. Flow cytometry for phagocytosis.

(**A** and **B**) Gating strategy for phagocytosis *in vitro* (A) and *in vivo* (B).

Table S1 Clinicopathological Features of HCC Patients Used in Immunoblotting Analysis

Accession number	Gender	Age (years)	Stage (BCLC)	Stage (CNLC)
850013	Male	48	**A**	**Ib**
519844	Male	67	**A**	**Ib**
866389	Male	58	**A**	**Ib**
855591	Male	41	**A**	**Ia**
867831	Male	68	**A**	**Ia**
874569	Female	71	**A**	**Ib**
874340	Female	61	**A**	**Ia**
864752	Male	59	**A**	**Ia**
869944	Female	42	**A**	**Ia**
871136	Female	56	**A**	**Ia**

Table S2 The Clinicopathological Characteristics of Liver Cancer Patients Enrolled in the Study

Location	Gender	Age (years)	pT status	pN status	pM status	TNM stage
A1	Male	33	1	0	0	I
A3	Male	50	1	0	0	I
A5	Male	67	1	0	0	I
A7	Male	44	1	0	0	I
A9	Male	42	1	0	0	I
A11	Male	66	1	0	0	I
A13	Male	38	1	0	0	I
A15	Female	59	1	0	0	I
B1	Male	58	1	0	0	I
B3	Male	73	1	0	0	I
B5	Male	59	1	0	0	I
B7	Female	74	1	0	0	I
B9	Male	57	1	0	0	I
B11	Male	51	1	0	0	I
B13	Male	52	1	0	0	I
B15	Male	31	1	0	0	I
C1	Male	52	1	0	0	I
C3	Male	43	1	0	0	I
C5	Male	36	1	0	0	I
C7	Male	58	1	0	0	I
C9	Male	55	1	0	0	I
C11	Male	31	1	0	0	I
C13	Male	43	1	0	0	I

C15	Male	39	1	0	0	I
D1	Male	43	1	0	0	I
D3	Male	34	1	0	0	I
D5	Male	44	2	0	0	II
D7	Male	27	2	0	0	II
D9	Male	75	2	0	0	II
D11	Male	58	2	0	0	II
D13	Male	30	2	0	0	II
D15	Male	70	2	0	0	II
E1	Male	50	2	0	0	II
E3	Male	67	2	0	0	II
E5	Male	35	2	0	0	II
E7	Male	51	2	0	0	II
E10	Male	58	2	0	0	II
E11	Male	49	2	0	0	II
E13	Male	43	2	0	0	II
E15	Male	47	2	0	0	II
F1	Male	48	2	0	0	II
F3	Male	65	2	0	0	II
F5	Male	53	2	0	0	II
F7	Female	48	2	0	0	II
F9	Male	48	3	0	0	III
F11	Male	32	3	0	0	III
F13	Male	39	3	0	0	III
F15	Male	37	3	0	0	III
G1	Male	44	3	0	0	III
G3	Male	40	3	0	0	III
G5	Female	51	3	0	0	III
G7	Male	63	3	0	0	III
G9	Female	58	3	0	0	III
G11	Male	55	3	0	0	III
G13	Male	39	3	0	0	III
G15	Male	47	3	0	0	III
H1	Male	53	3	0	0	III
H3	Male	54	3	0	0	III
H5	Male	53	3	0	0	III
H7	Male	62	3	0	0	III
H9	Male	65	3	0	0	III
H11	Male	46	4	0	0	III
H13	Male	48	3	0	0	III
H15	Male	54	2	1	1	IV
I1	Male	47	2	0	1	IV
I3	Male	41	2	0	1	IV

I5	Female	46	4	0	1	IV
I7	Male	43	2	0	1	IV
I9	Female	39	4	0	1	IV
I11	Male	39	4	0	1	IV
I13	Male	45	4	0	1	IV
I15	Male	60	4	0	1	IV
J1	Male	53	3	1	1	IV
J3	Male	55	3	1	1	IV
J5	Male	65	2	0	1	IV
J7	Male	56	4	0	1	IV
J15	Male	52	2	1	1	IV
A17	Male	46	1	0	0	I
C17	Male	62	1	0	0	I
E17	Male	47	1	0	0	I

Table S3 Sequences of the primers used for qRT-PCR

Gene	Primer sequence (5'→3')	Amplification size (bp)
HMMR	F: CAGTTGTCGAGGAGTGCCAG<br>R: GGTGCACAACCAGAAGGGT	107
CD47	F: GCGATTGGATTAACCTCCTTCGTCA<br>R: CCATGCATTGGTATACACGCCGC	113
tnfa	F: CTCGAACCCCGAGTGACAAG<br>R: TGAGGTACAGGCCCTCTGAT	159
ccl2	F: CAGCAGCAAGTGTCCCAAAG<br>R: CGGAGTTTGGGTTTGCTTGT	127
cxcl2	F: ATCAATGTGACGGCAGGGAAA<br>R: TCGAAACCTCTCTGCTCTAACAC	240
il-1b	F: GAGCAACAAGTGGTGTTCTCC<br>R: AACACGCAGGACAGGTACAG	110
cxcl3	F: AGCACCAACTGACAGGAGAG<br>R: TAAGTCCTTTCCAGCTGTCCC	137
GAPDH	F: CAAGCTCATTTCCTGGTATGAC<br>R: CAGTGAGGGTCTCTCTCTTCCT	142

Table S4 Reagents and antibodies

No	Catalog	Antibody	Sources	Dilution	Corp.
1	55463	HMMR	Rabbit	1:1000 (for WB)<br>1:100 (for IP)	Cell Signaling Technology
2	BS78352	HMMR	Rabbit	1:500 (for WB)	Bioworld Technology
3	Sc-515221	HMMR	Mouse	1:200 (for IF)	Santa Cruz

4	Ab124729	HMMR	Rabbit	1:200 (for IHC)	Abcam
5	3578	CD44	Rabbit	1:1000 (for WB)	Cell Signaling Technology
6	3570	CD44	Mouse	1:50 (for IP)	Cell Signaling Technology
7	Ab254530	CD44	Mouse	1:5000 (for IHC)	Abcam
8	Ab218810	CD47	Rabbit	1:1000 (for WB)<br>1:2000 (for IHC)	Abcam
9	ET1603-12	NF-κB p65	Rabbit	1:1000 (for WB)	HUABIO
10	YP0191	NFκB-p65 (phospho Ser536)	Rabbit	1:1000 (for WB)	Immunoway
11	Ab32360	NF-κB p105/p50	Rabbit	1:1000 (for WB)	Abcam
12	4806S	Phospho-NF-κB p105 (Ser932)	Rabbit	1:1000 (for WB)	Cell Signaling Technology
13	BS80104	IκBα	Rabbit	1:1000 (for WB)	Bioworld Technology
14	AP0707	Phospho-IκBα-S32	Rabbit	1:1000 (for WB)	Abclonal
15	AE005	anti DDDDK-Tag mAb	Mouse	1:1000 (for WB)<br>1:100 (for IP)	Abclonal
16	AE003	anti His-Tag mAb	Mouse	1:1000 (for WB)	Abclonal
17	71433	FAK	Rabbit	1:1000 (for WB)	Cell Signaling Technology
18	8556	Phospho-FAK (Tyr397)	Rabbit	1:1000 (for WB)	Cell Signaling Technology
19	2109	SRC	Rabbit	1:1000 (for WB)	Cell Signaling Technology
20	6943	Phospho-SRC (Tyr416)	Rabbit	1:1000 (for WB)	Cell Signaling Technology
21	5174	GAPDH	Rabbit	1:5000 (for WB)	Cell Signaling Technology
22	19589	CD86 Rabbit mAb	Rabbit	1:200	Cell Signaling Technology
23	Bs-1014R	Anti-CD11b Polyclonal Antibody	Rabbit	1:200	Bioss
24	Ab6640	Anti-F4/80	Rat	1:200	Abcam
25	557397	Anti-Mouse CD11b-PE	Rat	1:100	BD Biosciences
26	17-4081-82	F4/80 Monoclonal Antibody (BM8), APC	Rat	1:100	Invitrogen
27	563011	BD Horizon™ BV605 Rat Anti-Mouse Ly-6C	Rat	1:100	BD Biosciences
28	104743	Brilliant Violet 711™ anti-mouse CD80 Antibody	Hamster	1:100	Biolegend
29	563565	BD Horizon™ BUV395 Hamster Anti-Mouse CD3e	Hamster	1:100	BD Biosciences
30	103116	APC/Cyanine7	Rat	1:100	Biolegend

		anti-mouse CD45 Antibody			
31	612833	BD Horizon™ BUV737 Rat Anti-Mouse CD62L	Rat	1:100	BD Biosciences
32	561105	BD Pharmingen™ FITC Rat anti-Mouse Ly-6G	Rat	1:100	BD Biosciences
33	562291	BD Horizon™ PE-CF594 Rat Anti-Mouse CD19	Rat	1:100	BD Biosciences
34	740853	BD OptiBuild™ BV786 Mouse Anti-Mouse NK-1.1	Mouse	1:100	BD Biosciences
35	740455	BD OptiBuild™ BV650 Rat Anti-Mouse CD44	Rat	1:100	BD Biosciences
36	550954	BD Pharmingen™ PerCP-Cy™5.5 Rat Anti-Mouse CD4	Rat	1:100	BD Biosciences
37	552877	BD Pharmingen™ PE-Cy™7 Rat Anti-Mouse CD8α	Rat	1:100	BD Biosciences
38	560582	BD Pharmingen™ PE-Cy™7 Rat Anti-Mouse CD86	Rat	1:100	BD Biosciences
39	155309	Brilliant Violet 421™ anti-mouse CD163 Antibody	Rat	1:100	Biolegend
40	A-11034	Goat anti-Rabbit IgG (H+L) Highly Cross-Adsorbed Secondary Antibody, Alexa Fluor™ 488	Goat	1:500	Invitrogen
41	A-11037	Goat anti-Rabbit IgG (H+L) Highly Cross-Adsorbed Secondary Antibody, Alexa Fluor™ 594	Goat	1:500	Invitrogen
42	PKH26GL	PKH26 Red Fluorescent Cell Linker Kit		0.4 uM	Sigma Aldrich
43	C1031	CFSE		5 uM	Beyotime
44	C8160	Collagenase Type IV		1.5 mg/ml	Solarbio

45	C8140	Collagenase Type I		1.5 mg/ml	Solarbio
46	297-473-0	LPS		200 ng/ml	Sigma Aldrich
47	AF-315-05	IFN-γ		40 ng/ml	Peprotech
48	87788	Pierce™ IP lysis buffer			ThermoFisher SCIENTIFIC
49	C0065	DAPI solution			Solarbio
50	11668019	Lipofectamine™ 2000 Transfection Reagent			ThermoFisher SCIENTIFIC
51	HY-101053	SRC Inhibitor 1		2 uM	MedChemExpress
52	HY-10461	PF-573228		3 uM	MedChemExpress
53	S2913	BAY 11-7082		10 uM	Selleck

Uncropped gel for Fig. 6D.

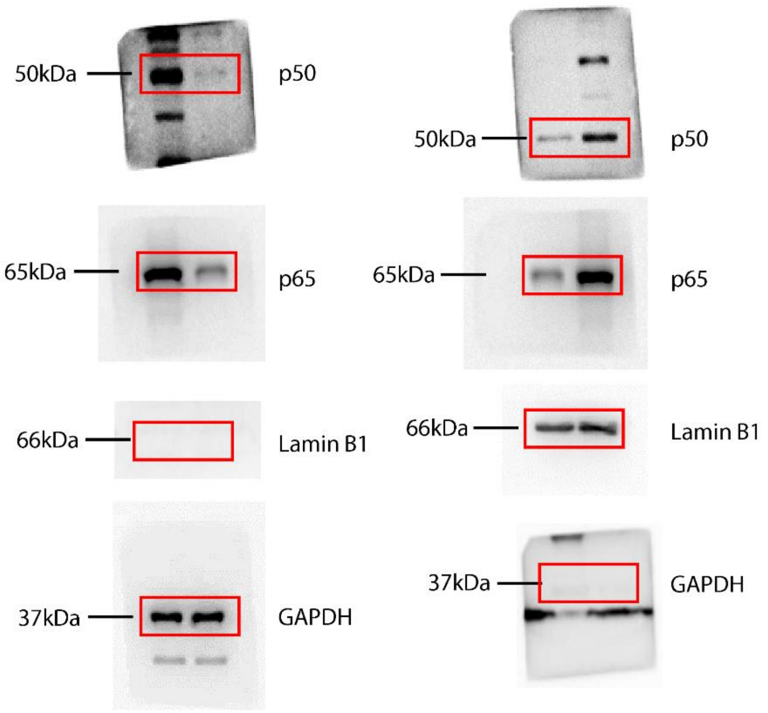

Supplement: Supplementary file 1 — Figs. S1 to S10 Tables S1 to S4 [file sciadv.adl6083_sm.pdf]
